# High-dimensional co-expression network analysis reveals persistent TRH gene expression throughout axolotl telencephalon regeneration

**DOI:** 10.3389/fbinf.2025.1697212

**Published:** 2026-01-12

**Authors:** Iveth Gómez-Morales, Adriana P. Mendizabal-Ruiz, J. Alejandro Morales, Teresa Romero-Gutiérrez

**Affiliations:** 1 Biodigital Innovation Lab. Centro Universitario de Ciencias Exactas e Ingenierías, Universidad de Guadalajara, Guadalajara, Jalisco, Mexico; 2 Farmacobiology Department, Centro Universitario de Ciencias Exactas e Ingenierías, Guadalajara, Jalisco, Mexico; 3 Centro Universitario de Tlajomulco, Universidad de Guadalajara, Tlajomulco de Zúñiga, Jalisco, Mexico

**Keywords:** axolotl, brain, hdWGCNA, hub genes, regeneration, single-cell, spatio-temporal, telencephalon

## Abstract

**Introduction:**

The Axolotl (*Ambystoma mexicanum*) offers a deep insight into brain regeneration by fully reconstructing its telencephalon post-injury, a capability that most vertebrates do not have. This study aimed to identify hub genes (highest-weighted genes) underlying this process and to map their cell location by analyzing spatiotemporal transcriptomic data using high-dimensional weighted gene co-expression network analysis, integrating protein-protein interaction networks, and cross-validating findings through literature.

**Results:**

We identified 180 hub genes across the regeneration timeline, including several with conserved orthologs previously reported in vertebrate regeneration models. Among these candidates, TRH (Thyrotropin-Releasing Hormone) displayed the most consistent spatiotemporal pattern, appearing repeatedly as a hub gene and localizing to MSN enriched regions at multiple stages. TRH is broadly characterized in vertebrates as a neuroendocrine peptide with roles in hormonal signaling, and MSNs are known to respond to a variety of hormonal and neuropeptidergic cues. In our dataset, this background provides additional perspective on the transcriptional configurations in which TRH appears. Other hub genes showed stage/cell specific patterns, together outlining a heterogeneous and dynamic landscape of transcriptional states detected during telencephalon regeneration.

**Conclusion:**

This study provides a descriptive map of gene co-expression dynamics during axolotl telencephalon regeneration. By integrating hdWGCNA, spatial transcriptomics, and network-based context, we identify hub genes and transcriptional states associated with injury response, including a persistent TRH linked MSN state. These findings offer a foundation for future experimental studies aimed at elucidating the molecular basis of axolotl brain repair.

## 1 Introduction

Studies on the regenerative abilities of salamanders, including brain tissue regeneration, have provided crucial insights into neuroregeneration (Joven et al., 2019; Arenas Gómez and Echeverri, 2021). Among salamander species, Axolotl is one of the most extensively studied models in developmental and regenerative biology (Arenas Gómez and Echeverri, 2021; Bölük et al., 2022). The axolotl’s capacity for brain repair offers a model to investigate the genetic regulatory mechanisms underlying neuro-regeneration through the analysis of hub genes identified via gene co-expression network analysis. In such networks, hub genes represent the central role of their modules and are recognized as central regulators of gene expression and cellular processes (Yu et al., 2017).

When facing disruptive events such as brain injury, organisms must reprogram cellular functions and molecular pathways to ensure tissue recovery. This process involves dynamic changes in cell-type proportions and gene regulatory programs that activate specialized regenerative mechanisms (Goldman and Poss, 2020; Bassat and Tanaka, 2021). Evidence of this phenomenon has been reported across diverse organisms, from highly regenerative species to mammals with limited capacity. In axolotls and newts, differentiated cells regain plasticity and contribute to tissue reconstruction, while zebrafish exhibit extensive regenerative potential in organs such as the heart and fins through dynamic cellular remodeling. Planarians regenerate entire bodies by modulating the abundance of pluripotent cells, and Hydra maintains continuous self-renewal through flexible transcriptional programs. Partial manifestations of this process have also been documented in mammals, particularly in liver regeneration and neonatal cardiac repair (Gerber et al., 2018; Kikuchi et al., 2010; Fincher et al., 2018; Siebert et al., 2019; Yanger et al., 2013; Li et al., 2021).

Notably, a recent study employed surgical removal of the telencephalon in the axolotl, monitoring regeneration with single-cell spatial transcriptomics (Wei et al., 2022), Stereo-seq, the technology used to generate this dataset, is a sequencing-based spatial transcriptomics platform that captures mRNA using patterned DNA nanoball arrays and achieves subcellular to near–single-cell resolution (Chen et al., 2022). The dataset analyzed here was processed with the Stereo-seq Analysis Workflow (SAW), which performs read alignment, spatial binning, and segmentation to produce gene-by-cell matrices suitable for downstream analysis (Zhang and Horvath, 2005).

Here, we introduce a framework to elucidate the spatiotemporal gene regulatory landscape of axolotl brain regeneration by focusing on hub genes as functional proxies for cell-type specific programs. We present an integrative framework that leverages stereo-seq transcriptomic data and high-dimensional weighted gene co-expression network analysis (hdWGCNA). This approach identifies co-expressed gene modules across distinct cell-types and the extraction of eigengenes, summary profiles that capture the core activity of each module. By constructing eigengene networks and pinpointing hub genes with the highest connectivity, we map candidate regulatory drivers within and between neighboring cell populations during regeneration.

This systems-level approach deepens our understanding of the molecular mechanisms underlying brain repair in the axolotl and lays the groundwork for developing novel therapeutic strategies to promote regeneration and recovery following severe central nervous system injury.

## 2 Materials and methods

This study aimed to identify and characterize key regulatory hub genes involved in telencephalon regeneration in the axolotl by leveraging spatial transcriptomic datasets and computational network analysis. This research was designed as a retrospective bioinformatics study utilizing publicly available spatial transcriptomic data derived from axolotl telencephalon tissue across multiple regenerative and control stages.

### 2.1 Spatial transcriptomics dataset

Spatial transcriptomic data from the telencephalon of Axolotl were retrieved from the public STOmics/ARTISTA repository (https://db.cngb.org/stomics/artista/), corresponding to the dataset reported by Wei et al. (Science, 2022). This dataset comprises 24 telencephalic sections spanning developmental stages (St. 44, 54, 57), juveniles, adults, metamorphosed individuals, and post-injury samples collected at 2, 5, 10, 15, 20, 30, and 60 days post-injury (DPI). Telencephalic injury experiments in the original study were performed in juvenile axolotls (10–13 cm in body length), in which brain lesions were generated by controlled extirpation of a reproducible 0.5 × 0.5 mm portion of the dorsal pallium in the left telencephalic hemisphere of ∼11 cm individuals.

In the original publication, raw Stereo-seq reads were initially processed using the SAW (Stereo-seq Analysis Workflow), which performs read alignment, spatial registration, single-cell segmentation, and quantification of unique molecular identifiers (UMIs) to generate cell-by-gene expression matrices. The resulting matrices, deposited in STOmics, are fully preprocessed, quality-controlled, and annotated to the Axolotl reference genome (AmexG_v6.0-DD, axolotl-omics.org). The genome annotation was generated by the genome authors based on sequence homology to orthologous genes from other vertebrates, primarily human. Consequently, the dataset includes both genes labeled with human-like symbols and Axolotl specific identifiers (e.g., AMEX60DD_XXXXX). All genes present in the preprocessed matrices were retained to capture both conserved and axolotl-specific transcripts.

For the present study, analyses were restricted to juvenile, adult, and post-injury telencephalic sections, excluding all developmental-stage and metamorphosed samples. We analyzed all injury-associated sections (2–60 DPI) and used uninjured juvenile and adult samples as biological controls. Preprocessed matrices and corresponding metadata were downloaded in RDS format and imported into R (v4.2.2) using the native readRDS() function. No additional preprocessing, filtering, or realignment was applied, thereby preserving the original metadata, spatial coordinates, and gene annotations for downstream transcriptomic and co-expression analyses.

### 2.2 Library and requirements

All analyses were conducted in RStudio using Seurat v4.4 (https://github.com/satijalab/seurat) and hdWGCNA v0.4.01 (https://smorabit.github.io/hdWGCNA/) as the primary toolkit for spatial and single-cell transcriptomic analyses. Additional dependencies, including WGCNA, UCell, GenomicRanges, GeneOverlap, and devtools were installed to support gene network construction, module comparison, and spatial mapping. The Seurat package was installed directly from the satijalab GitHub repository to ensure compatibility with hdWGCNA workflows.

### 2.3 Co-expression network construction

Weighted gene co-expression networks were built for each regeneration time point (2, 5, 10, 15, 20, 30, and 60 DPI) and for both control conditions (juvenile and adult) using the hdWGCNA framework, following the official workflow for single-cell data. All analyses were performed independently for each annotated cell-type within each sample, as implemented in the supplementary code *hdWGCNA_pipeline.R*.

Gene filtering was conducted using the SetupForWGCNA() function with *gene_select = “fraction”* and a detection threshold of 5% (*fraction = 0.05*). Under this configuration, a gene is retained only if it is expressed in at least 5% of cells within each specific cell-type. This parameter is defined by the hdWGCNA framework and is applied directly to the data; consequently, genes with sparse or highly cell-type restricted expression may be excluded from network construction in cell-types where they do not meet this threshold. Expression values were extracted from the SCTransform-normalized assay (“SCT”, slot = “counts”), which contains Pearson residuals generated through regularized negative binomial regression. These variance stabilized values preserve biological heterogeneity while mitigating batch effects and sequencing depth variation.

Metacell aggregation was used to increase network stability and reduce noise. Metacells were generated with MetacellsByGroups() by grouping cells according to their annotated identity (*group.by = “Annotation”*) and aggregating *k = 25* nearest neighbors per metacell (with *max_shared = 30*). The resulting metacell matrices were normalized using NormalizeMetacells() before network construction.

Soft-thresholding power was estimated for each cell-type using TestSoftPowers() across the default candidate range (1–30). hdWGCNA selects the lowest power that achieves a scale-free topology fit index (R^2^ ≥ 0.80), ensuring adequate network connectivity and adherence to scale-free properties. When no candidate power reached this threshold for a given cell-type, that cell-type was excluded from downstream network analysis. This exclusion results from framework defined criteria, and not from gene expression patterns alone.

Network construction was then performed with ConstructNetwork() using the selected soft power for each cell-type. Module eigengenes (MEs) were computed using ModuleEigengenes(), scaled with ScaleData() while preserving cell-type annotation, and stored within the Seurat object. Gene-module connectivity (kME) was obtained with ModuleConnectivity() to quantify the correlation between each gene and its module eigengene, enabling the identification of highly connected hub genes.

### 2.4 Identification of regeneration-expressed genes

To identify genes exhibiting regeneration-specific expression patterns, we employed a comparative set-based strategy integrating co-expression modules from hdWGCNA outputs across all timepoints and conditions (‘common_genes_across_timepoints.R’).

All genes assigned to co-expression modules were extracted from hdWGCNA results and compiled into a comprehensive gene by cell-type matrix stratified by timepoint (Supplementary Data Sheet 1). Gene set comparisons were performed using set intersection operations to identify genes with consistent module membership across conditions. This analysis generated three derivative datasets: 1. genes present across all seven regeneration stages (2–60 DPI; Supplementary Data Sheet 2); 2. genes present in both control samples (juvenile and adult; Supplementary Data Sheet 3); and 3. regeneration-specific genes present in regeneration but absent from control samples (Supplementary Data Sheet 4).

Genes detected during regeneration were first partitioned according to their annotation status. AMEX-prefixed identifiers were separated from genes with established ortholog assignments, and both categories were retained for analysis in distinct output files. Ortholog names were standardized by removing taxonomic suffixes and extracting gene symbols from the pipe delimited format.

For the purposes of this study, genes of interest were defined as those assigned to co-expression modules present in regenerating samples (2–60 DPI) and absent from modules identified in control tissues. This classification is based on the assumption that co-expression modules reflect coordinated transcriptional activity, enabling the identification of gene programs specifically associated with the post-injury context.

### 2.5 Hub gene extraction and frequency analysis

For each regeneration timepoint, hub genes were extracted from hdWGCNA network outputs using GetHubGenes() with n_hubs = 10, identifying the top 10 most highly connected genes (by kME) within each co-expression module for every cell-type (hubgenes_analysis.R). Hub genes were ranked by their module connectivity, assigning rank levels 1–10 where level 1 represents the most central hub gene.

The 180 regeneration-associated genes identified were cross-referenced with hub gene lists to determine in which cell-types, modules, and at what rank level each gene appeared. Genes not identified as hub genes were filtered out, retaining only hub genes for downstream spatial analysis.

To quantify how frequently each regeneration gene functioned as a hub across different cellular contexts, module assignments were parsed and counted using in hubgene_getfrequencie.R.

This frequency analysis revealed which regeneration genes consistently emerged as network hubs across modules and timepoints. Hub gene annotations including AMEX gene identifiers, gene names, associated cell-types, modules, rank positions, and hub frequencies were consolidated in Supplementary Data Sheet 5.

### 2.6 Spatial mapping of cell-types and hub gene expression

Spatial transcriptomics data were processed using Seurat’s standard preprocessing workflow (hdWGCNA_spatial_pipeline.R), which included normalization, identification of highly variable features, data scaling, and principal component analysis. Shared nearest neighbor graphs were constructed using the first 30 principal components, followed by UMAP dimensionality reduction.

Spatial visualization of cell-type distributions was performed with annotation based grouping. Visualization parameters were optimized for clarity, coordinate transformation, and a custom 40 color palette for distinct cell-type identification. Spatial maps were generated individually for each regeneration timepoint by manually loading the corresponding spatial Seurat object (RDS file).

For spatial expression analysis of hub genes, each of the 180 regeneration associated hub genes was manually queried across all regeneration timepoints using spatial transcriptomics datasets. The analysis prioritized genes at rank 1 within their respective cell-types and modules. Spatial expression patterns were retrieved from Seurat object metadata and visualized manually, constructing a spatiotemporal atlas of hub gene expression throughout the temporal progression of brain regeneration.

### 2.7 STRING

Protein–protein interaction (PPI) networks were generated using STRING (accessed August 2024) to explore potential functional relationships among the 180 hub genes identified across regeneration time points. The original annotation relies on the Axolotl reference genome (AmexG_v6.0-DD, axolotl-omics.org), which integrates gene symbols inferred through cross-species homology.

For this analysis, we used the gene symbols provided in that original annotation, which were assigned based on best-hit homology to the human proteome and serve as functional proxies for axolotl genes.

The *Xenopus laevis* proteome was selected as the reference organism in STRING due to its phylogenetic proximity to Axolotl within Amphibia, allowing accurate orthology-based transfer of conserved protein interactions while preserving lineage-specific context. This approach minimizes annotation mismatches that may occur when querying against mammalian databases. All evidence channels in STRING were enabled, and pairwise associations with a combined confidence score ≥0.40 were retained for network construction and visualization.

### 2.8 Spatial co-localization analysis of TRH expression and MSN cells during regeneration

To investigate the spatial distribution of TRH (Thyrotropin-Releasing Hormone) expression and its topographical relationship with MSN (Medium Spiny Neurons) populations throughout axolotl brain regeneration, spatially-resolved gene expression maps were retrieved from the ARTISTA database in PNG format for each control and post-injury time point examined (2, 5, 6, 10, 15, 21, 30, and 60 DPI). For each one, two distinct image datasets were acquired: one depicting TRH expression within the lesioned tissue and another representing MSN cell distribution in the corresponding region. Image processing was performed using a custom Python script implementing a color based detection and morphological dilation algorithm. The algorithm identifies expression signals in each image through intensity thresholding in the RGB color space, applies binary dilation to enhance marker visibility, and generates a composite image wherein TRH expression is rendered in grayscale and red gradients according to signal intensity, while MSN cell presence is encoded in yellow. Signal overlay enables the identification of spatial co-localization regions where TRH expression occurs in proximity to or within MSN neuronal territories. The resulting composite images were exported at 300 DPI resolution with transparent backgrounds, facilitating visualization of temporal changes in TRH distribution patterns and its spatial association with MSN cells throughout the regenerative process.

## 3 Results

### 3.1 Hierarchical filtering of co-expression networks defines a regeneration-associated hub gene set

**FIGURE 1:**
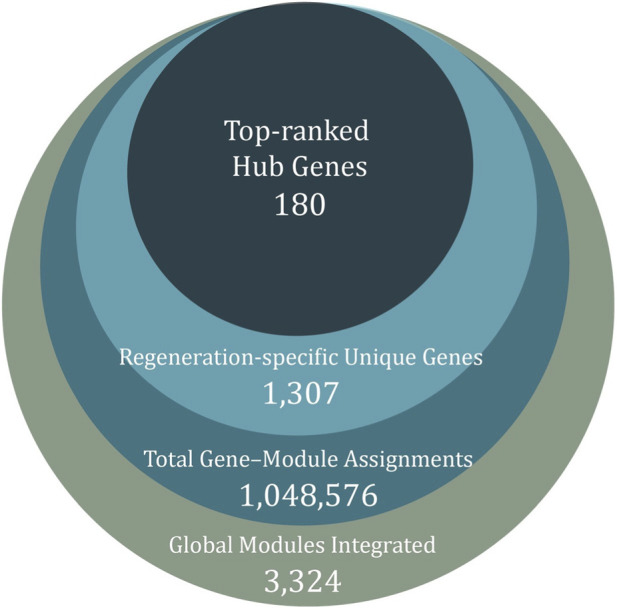
Hierarchical filtering of gene co-expression data. The outer circle represents all gene–cell entries (1,048,576) grouped into 3,324 co-expression modules. From these, 1,307 genes were identified as specific to regeneration, and 180 hub genes were retained as representative regulators.

We first summarized the global co-expression structure of the dataset by constructing hdWGCNA networks across all cell-types, regeneration stages and uninjured controls. This analysis yielded 1,048,576 gene–cell assignments distributed into 3,324 co-expression modules, each representing a group of genes with shared expression patterns in a specific cellular and temporal context (Figure 1). A given gene may appear multiple times if it is detected in different modules across timepoints or cell-types, whereas genes not assigned to any module are grouped in the “grey” category and excluded from downstream interpretation.

To focus on genes associated with the post-injury response, we compared module composition between regenerating samples and controls. Genes present in control modules were excluded. This yielded 1,307 genes restricted to regeneration-specific modules, comprising both annotated orthologs and axolotl-specific AMEX-prefixed genes. Of these, 1,237 correspond to genes with assigned orthologs, while the remaining AMEX-labeled genes represent axolotl-specific transcripts that were used for future analysis separately.

Within this regeneration-restricted set, we then examined intramodular connectivity to identify hub genes as the most highly connected members of each module. Aggregating hub assignments across all regeneration-associated modules yielded a non-redundant set of 180 hub genes. This hub gene ensemble constitutes the core group of candidates analyzed in subsequent sections, where we characterize their temporal dynamics, spatial distribution and interaction context.

### 3.2 Global distribution of hdWGCNA modules across the regeneration timeline

**FIGURE 2:**
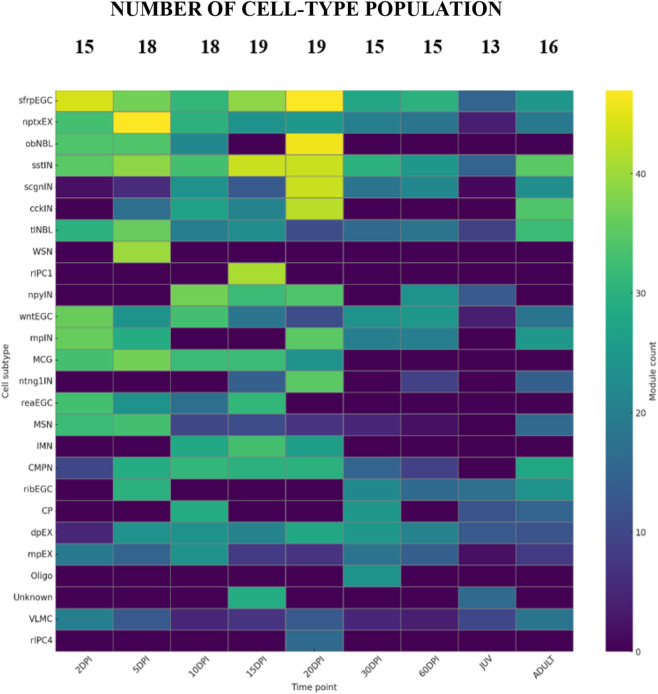
Global distribution of hdWGCNA co-expression modules during axolotl telencephalon regeneration. Heatmap showing the number of hdWGCNA modules detected per cell subtype across stages (uninjured juvenile/adult; 2, 5, 10, 15, 20, 30, and 60 DPI). Numbers above indicate total cells per stage. Values are aggregated across subtypes; detailed subtype contributions are provided in Supplementary Data Sheet 2. Abbreviations: sfrpEGC, Sfrp + Ependymoglial; nptxEX, Nptx + lateral pallium excitatory neurons; obNBL, olfactory bulb neuroblasts; sstIN, Sst + inhibitory neurons; scgnIN, Scgn + inhibitory neuron; cckIN, Cck + inhibitory neurons; tlNBL, telencephalon neuroblasts; WSN, wound-stimulated neurons; rIPC1, reactive intermediate progenitor cells 1; npyIN, Npy + inhibitory neuron; wntEGC, Wnt + ependymoglial cells; mpIN, medial pallium inhibitory neurons; MCG, Microglia; ntng1IN, Ntng1+ inhibitory neurons; reaEGC, reactive ependymoglial cells; MSN, medium spiny neuron; IMN, immature neurons; CMPN, cholinergic/monoaminergic/peptidergic neurons; ribEGC, ribosomal EGC; CP, choroid plexus; dpEX, dorsal pallium excitatory neurons; mpEX, medial pallium excitatory neuron; Oligo, oligodendrocytes; VLMC, leptomeningeal vascular cells; rIPC4, reactive intermediate progenitor cells 4.

To obtain a systems-level view of how transcriptional programs evolve over time, we next summarized the global distribution of hdWGCNA modules across all analyzed cell subtypes and regeneration stages in a heatmap displayed in Figure 2. The total number of co-expression modules detected when aggregating all cell-types. The X-axis represents the full regeneration timeline, including uninjured controls and post-injury stages at 2, 5, 10, 15, 20, 30, and 60 DPI. The Y-axis lists the annotated cell-types (clusters) present in the dataset. Each tile represents the number of modules detected within a specific cell-type at each timepoint.

The numbers shown above the heatmap indicate the total number of cells analyzed at each timepoint, enabling visual comparison between module counts and sampling depth. Detailed information on cell-type annotations and their presence across the regeneration timeline is provided in Supplementary Data Sheet 2.

This representation shows that multiple cell-types display a higher number of co-expression modules at early and intermediate post-injury stages compared with uninjured controls. By contrast, at later timepoints (30–60 DPI), module counts within most cell-types converge toward values similar to those observed in controls. Given this dynamic and complex modular landscape, we focused subsequent analyses on hub genes allowing us to summarize co-expression programs at single-cell resolution.

### 3.3 Cell-level mapping of hub genes reveals regeneration-specific regulatory signatures

**FIGURE 3:**
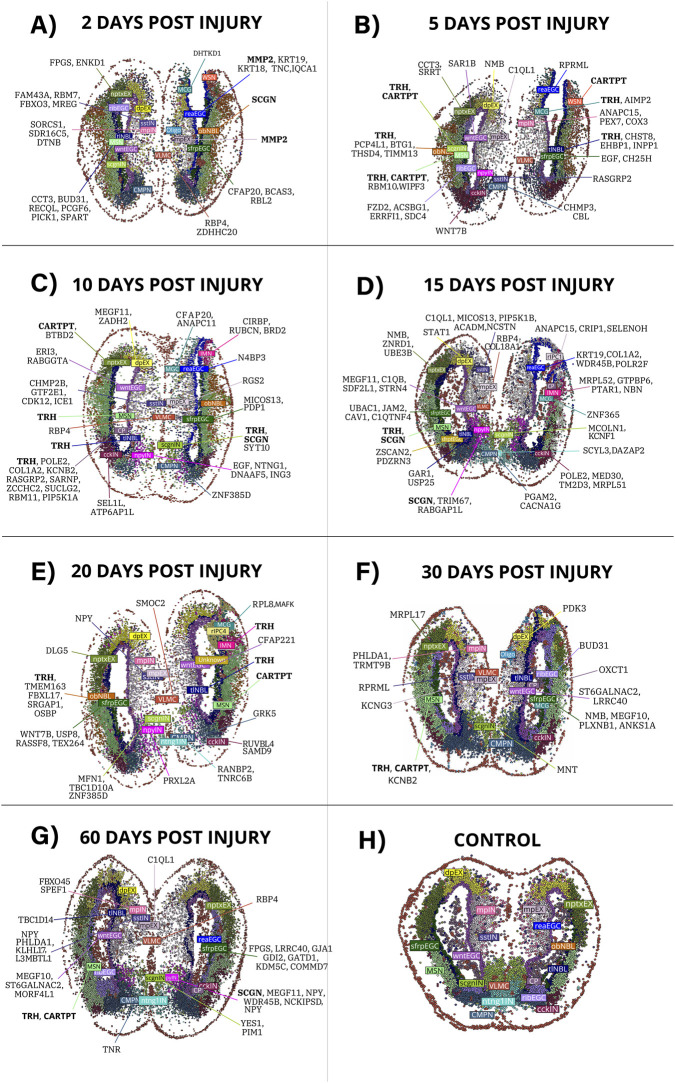
Hub genes detected in individual cells across post-injury stages (2, 5, 10, 15, 20, 30, and 60 DPI) and in uninjured controls. **(A–G)** display the distribution of hub genes identified by hdWGCNA in each cell across the corresponding regeneration time points. **(H)** shows the control condition, where no regeneration-associated hub genes were detected, reflecting that the hub genes identified in this study were specific to post-injury co-expression modules and did not appear in the uninjured telencephalon.

Given this dynamic modular landscape across regeneration stages, we examined how individual hub genes are distributed at single-cell resolution along the regeneration timeline. For each cell, the hdWGCNA networks were used to identify the top-ranked hub gene (highest kME) within its assigned module. Figure 3 summarizes these assignments by displaying, for every cell, the hub gene with the strongest intramodular connectivity at post-injury stages and in uninjured controls. To support the biological interpretation of these 180 prioritized hubs, Supplementary Data Sheet 6 provides a curated table compiling reported gene functions and injury/regeneration-relevant evidence for each hub gene, with the corresponding bibliographic sources.

Each panels A–G of Figure 3 shows the distribution of hub genes in single cells at 2, 5, 10, 15, 20, 30, and 60 DPI, respectively. Each panel displays the top-ranked hub gene within each cell’s assigned module. This representation allows the spatial and cellular distribution of hub-based regulatory states to be compared across timepoints.

Panel H displays the uninjured control condition, where none of the hub genes from regeneration-specific modules were detected as the top-ranked hub in any cell. This absence reflects the filtering used to define the 180 hub genes, which were selected from modules present only in post-injury samples and therefore do not appear in control modules by construction. Thus, the hub genes analyzed here are observed exclusively in post-injury conditions and are not characteristic of the baseline uninjured state.

Within this regeneration-associated hub gene set, several genes showed recurrent detection across multiple cell-types and regeneration stages (Supplementary Data Sheet 5). Notably, TRH (Thyrotropin-Releasing Hormone) emerged as one of the most frequently assigned hub genes, appearing as the top-ranked hub in multiple modules and across several cell-types, including medium spiny neuron–like populations, throughout the post-injury timeline. In addition, approximately 30 other hub genes were identified as hubs in more than one module and cell-type, showing that a subset of genes repeatedly occupies central positions in distinct co-expression programs during telencephalon regeneration.

### 3.4 Protein-protein interaction network organization of regeneration-associated hubgenes

**FIGURE 4:**
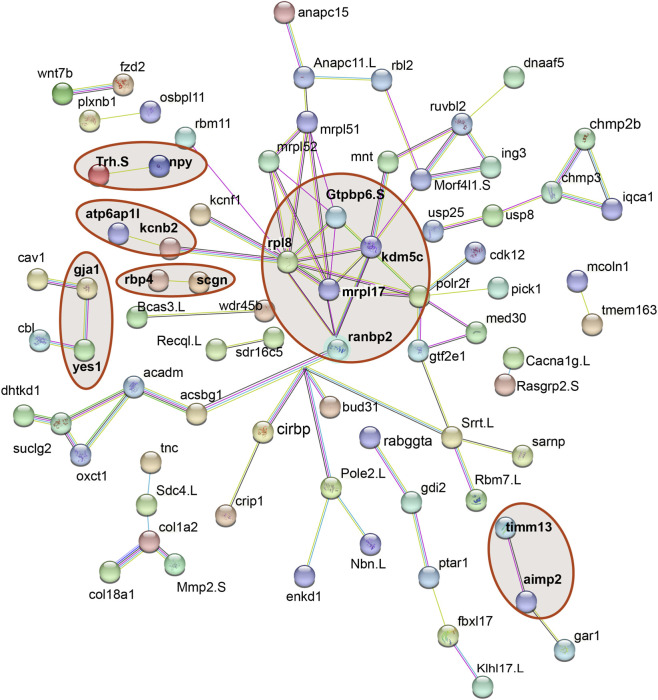
Protein–protein interaction (PPI) network of 180 hub genes identified from Axolotl transcriptomic data. Gene symbols were annotated via homology to human genes and queried in STRING using *Xenopus laevis* as the reference species, given the absence of axolotl-specific PPI data (Supplementary Data Sheet 7). Blue lines are known interactions from curated databases and pink lines are known interactions from experimentally determined. Green, red and navy blue lines correspond to predicted interactions from gene neighbourhood, gene fusions, gene co-occurrence. Yellow, black and purple lines correspond to textmining, co-expression, protein homology respectively.

Beyond their spatial and cellular distribution, we examined potential functional relationships among the 180 hub genes by constructing a protein–protein interaction (PPI) network using STRING (Figure 4). Because axolotl-specific PPI resources are not available, gene symbols were first mapped to their human orthologs in the original annotation and then queried in STRING using *Xenopus laevis* as the reference organism, thereby leveraging conserved vertebrate interaction information. A complete record of all 180 input genes and the corresponding *X. laevis* identifiers used for the STRING query is provided in Supplementary Data Sheet 7.

The resulting network depicts the interaction landscape among the proteins encoded by the 180 hub genes. Blue edges represent interactions curated from databases and pink edges correspond to experimentally determined interactions. Green, red and navy blue lines indicate predicted associations based on gene neighborhood, gene fusions and gene co-occurrence, respectively, whereas yellow, black and purple lines reflect relationships supported by text mining, co-expression and protein homology.

The PPI network reveals 180 hub genes connected by 523 interactions, with clustering of genes into distinct subnetworks. Some genes show extensive connectivity (>10 interactions), while others have limited connections (1-3 interactions). In the context of limited axolotl-specific functional annotation, this network provides a structural overview of how regeneration-associated hub genes are organized within known and predicted vertebrate interaction pathways.

### 3.5 Spatial mapping of TRH-expressing cells during telencephalon regeneration

**FIGURE 5:**
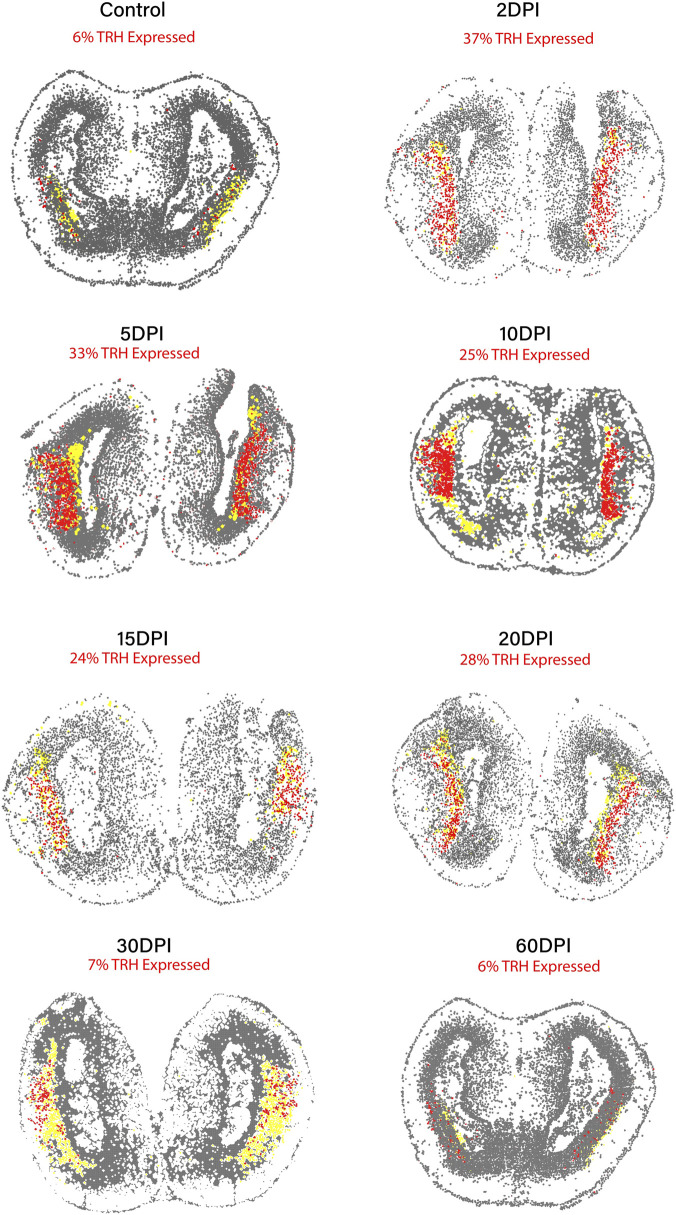
Spatial distribution of TRH-expressing cells across the regeneration timeline in the axolotl telencephalon (2–60 DPI) and Control (Juvenile). Spatial transcriptomic maps showing TRH-expressing cells (red) overlaid on medium spiny neuron (MSN) annotations (yellow) at multiple post-injury stages. Grey dots represent all spatially resolved cells. TRH-positive signals consistently localize within MSN-enriched regions from early (2 DPI) to late regeneration (60 DPI), illustrating the persistence of TRH-associated MSN states throughout the regenerative process. This visualization is descriptive and does not imply functional involvement of TRH in regeneration.

Given that TRH emerged as one of the most recurrent hub genes in regeneration-associated modules, we next examined its spatial distribution and temporal dynamics across the telencephalon. TRH-expressing cells were overlaid on MSN annotations using Stereo-seq spatial coordinates for each post-injury stage and for the juvenile control (Figure 5). In these maps, grey dots represent all spatially resolved cells, yellow indicates MSN-annotated cells, and TRH-positive cells are shown in red.

Quantitatively, TRH-positive cells accounted for approximately 6% of all cells in the juvenile control and increased markedly after injury, reaching 37% at 2 DPI and 33% at 5 DPI. This proportion then gradually decreased to 25% at 10 DPI, 24% at 15 DPI, and 28% at 20 DPI, and subsequently declined to 7% at 30 DPI and 6% at 60 DPI, approaching control levels at later stages. These percentages refer to the fraction of TRH-positive cells relative to the total cellular population in each section, not exclusively to the MSN subset.

Across the entire regeneration timeline (2–60 DPI), TRH-positive cells are predominantly located within MSN-enriched regions. These spatial and quantitative observations show that TRH expression is transiently expanded after injury and remains repeatedly associated with MSN-enriched areas throughout the post-injury period, in agreement with its recurrent identification as a hub gene in MSN linked modules. This visualization is intended as a descriptive summary of TRH dynamics and co-localization and does not, by itself, establish any functional contribution of TRH to the regenerative process.

## 4 Discussion

We used hdWGCNA and spatial transcriptomics to map transcriptional states across axolotl telencephalon regeneration, identifying 180 hub genes that characterize the regenerative timeline. TRH emerged as the most consistent hub, appearing in multiple co-expression modules and showing persistent spatial overlap with MSN-enriched regions from 2 to 60 DPI. Prior vertebrate studies have associated TRH with molecularly defined neuronal subpopulations and neuroprotective effects (Boutej et al., 2017; Maness, 1992). In this context, our integrative hdWGCNA spatial framework places TRH within a spatially resolved regenerative timeline; however, it cannot resolve whether TRH contributes causally to regeneration or primarily marks an injury-responsive transcriptional state.

Understanding hub genes in regenerative contexts requires methods that preserve cellular heterogeneity. hdWGCNA addresses this by constructing co-expression networks at single-cell resolution, avoiding the information loss inherent to bulk or pseudo-bulk approaches. This method has successfully identified biologically relevant hub genes in Alzheimer’s disease, tumor microenvironments, and tissue degeneration, contexts where cellular diversity drives pathology. In axolotl brain regeneration, where marked spatial and temporal heterogeneity defines the process, this single-cell resolution becomes particularly valuable (Morabito et al., 2023; Sun et al., 2024; Zhao et al., 2025; Zhu et al., 2025; Zhai et al., 2025).

In our analysis, hub genes represent the most highly connected nodes within cell-type specific modules, serving as proxies for dominant transcriptional programs. We focus here on the subset of hubs that appear specifically in regeneration (absent from controls), as these are most likely to mark injury-responsive states. The 180 hubs identified span all regeneration stages and multiple cell-types, enabling both spatiotemporal mapping and cross-species comparison.

Among the 180 hub genes identified, 104 have orthologs previously implicated in regenerative processes across *Danio rerio*, *Xenopus laevis*, *Ambystoma mexicanum*, and *Hydra* spp. (Supplementary Data Sheet 6), This overlap is consistent with the view that at least part of the molecular machinery engaged during axolotl telencephalon regeneration involves genes that have been repeatedly associated with regeneration in other metazoan models, within coordinated modules mediating intercellular communication and tissue reorganization (Goldman and Poss, 2020; Bassat and Tanaka, 2021; Gerber et al., 2018; Kikuchi et al., 2010; Fincher et al., 2018; Siebert et al., 2019; Yanger et al., 2013; Wei et al., 2022). The remaining 76 hubs include both conserved vertebrate genes and axolotl-specific transcripts (AMEX-prefixed identifiers), reflecting a combination of shared components and responses that appear restricted to this species.

Taken together, these patterns suggest that the telencephalic response in this species involves genes repeatedly associated with regeneration in other models alongside additional components without previously described regenerative roles, which we interpret as a descriptive correspondence at the level of gene identity rather than evidence of conserved function across species.

Within this mixed repertoire of conserved and axolotl restricted hubs, several genes also exhibit context-dependent transcriptional roles, whereby the same gene participates in distinct co-expression programs across cellular states. MMP2 illustrates this pattern by functioning as a hub in both reaEGC and mpEX cells during regeneration, within cell-type resolved co-expression modules. In mammals CNS injury, MMP2 and TNC are both engaged in wound healing remodeling and axonal regeneration (Hsu et al., 2006; Chen et al., 2010). The appearance of MMP2 in reaEGC alongside TNC, and its detection in other populations expressing SCGN, places these cell-types within a shared transcriptional context during early tissue remodeling. Consistent with this link, secretagogin has been mechanistically connected to neuronal MMP2 externalization in mammalian migratory circuits (Hanics et al., 2017) SCGN has also been linked to neuronal function and synaptic activity (Tu et al., 2023), and in other secretory systems its downregulation has been associated with increased vulnerability to stress induced cell death (Ouyang et al., 2024).

Transient immune populations also emerged specifically in the post-injury context. MCG were detected at 2 DPI and largely declined by 30 DPI (Figures 2, 3), paralleling the acute-to-resolving dynamics described after mammalian traumatic brain injury, where early microglial activation can support neuronal survival and tissue repair, whereas failure to resolve contributes to chronic neuroinflammation and secondary neurotoxicity (Loane and Kumar, 2016). TRH emerged as a hub gene within MCG at 5 DPI (Figure 3B); however, TRH-positive cells were already observed from 2 DPI in the spatial analysis (Figure 5), temporally aligning with this acute phase. Rather than implying microglial TRH production, we interpret this signal in the context of hypothalamic injury metabolic circuits where microglial inflammatory states can impact neighboring TRH neurons and systemic thyroid axis output (Veronesi et al., 2007). Consistent with this interpretation, TRH and TRH analogs have documented neuroprotective actions in vertebrate nervous system models, including protection against excitotoxicity/glutamate-induced toxicity, oxidative stress, and inflammatory injury (Daimon et al., 2013; Kim et al., 2024). Whether the TRH-linked microglial program actively modulates inflammation or reflects coordinated cross-talk with other TRH expressing populations remains unresolved; however, thyroid hormone signaling has been shown to shape microglial immune responsiveness and to regulate adult neural stem/progenitor dynamics in injury-relevant contexts (López-Juárez et al., 2012; Thorrez et al., 2008).

Unlike hubs with clear functional annotation, we also observed a transient translation/ribosome associated hub signature, exemplified by the 60s ribosomal protein RPL8 emerging as a hub exclusively at 20 DPI within a microglia-enriched module (Figure 3E). Ribosomal protein transcripts are frequently modulated by global transcriptional output and cell-state transitions, and their coordinated variation can influence co-expression network structure even when they are not specific effectors of a regenerative pathway (Hafemeister and Satija, 2019; Ni et al., 2025; Boutej et al., 2017). This is consistent with single-cell RNA-seq normalization frameworks that treat ribosomal genes as a major source of structured variation when modeling cell-to-cell expression differences (Ni et al., 2025). In microglia, activation states also involve post-transcriptional checkpoints that reshape translation programs, further supporting the interpretation of transient ribosome-linked hubs as markers of dynamic immune states rather than direct regenerative drivers (Szklarczyk et al., 2023). To place this hub signal in an orthogonal context, we queried STRING as a hypothesis generating resource. STRING linked RPL8 to GTPBP6, MRPL17, and KDM5C (Figure 4), genes detected in transitional populations at later stages (15–60 DPI; Figures 3D–G); however, these edges reflect curated and predicted associations and do not establish coordinated activity across regeneration (Hanics et al., 2025). Notably, regulatory nodes such as RANBP2 have been implicated in selective translational control in neural systems, supporting the possibility that ribosome-associated signals can emerge during state remodeling without implying direct regenerative function.

In contrast to transiently detected hubs like RPL8, SCGN showed more sustained and interpretable neuronal signature. SCGN appeared as a hub across multiple neuronal populations, including scgnIN at 10 DPI, npyIN, MSN at 15 DPI and npyIN again at 60 DPI (Figures 3C,D,G). During mammalian forebrain development, SCGN marks subsets of developing cortical GABAergic neurons and its expression is activity-modulated (Alzu’bi and Clowry, 2020; Raju et al., 2018; Maness, 1992), processes essential for circuit reconstruction. The detection of SCGN in inhibitory interneurons (npyIN) at both mid and late regeneration stages, alongside its appearance in MSN populations at 15 DPI, may reflect involvement in neuronal maturation programs across multiple cell-types. At late stages (60 DPI), additional hubs emerged in non-neuronal populations, including YES1 in scgnIN and GJA1 in sfrpEGC cells, both implicated in neuronal development and glial homeostasis in mammalian systems (Moore and O’Brien, 2015; Muñoz-Manchado et al., 2018).

Together, this sustained interneuron associated hub signatures motivated us to examine whether any candidate showed a similarly coherent pattern across the MSN enriched compartment over the full regenerative timeline.

Among all hub genes identified in our dataset, TRH showed the most consistent spatiotemporal pattern, appearing as a hub across multiple stages and maintaining persistent spatial association with MSN-enriched regions from 2 to 60 DPI (Figure 5). This persistence distinguishes TRH from other hubs that showed more restricted temporal or cellular distributions. However, the TRH-MSN association detected here likely represents an injury-induced transcriptional state rather than a canonical MSN identity. In healthy mammalian striatum, TRH expression is typically confined to interneuron lineages and absent from MSNs, (Cantuti-Castelvetri et al., 2010), yet Parkinsonian rats treated with chronic L-DOPA show TRH induction specifically in dorsal striatal MSNs and their projections (Zhu et al., 2024), demonstrating that MSN TRH expression can be state-dependent and context-engaged. Mechanistically, TRH can act on striatal GABAergic neurons, preferentially D2-MSNs, through a TRHR-MAPK-RARα-DRD2 pathway that modulates MSN phenotype (Obukohwo et al., 2024). Whether this mechanism operates in axolotl regeneration, or whether TRH expression simply accompanies the injury-responsive state without active signaling involvement, cannot be determined from transcriptomic data alone.

Beyond individual gene patterns, this work provides a spatiotemporal reference of hub gene activity across axolotl telencephalon regeneration. The spatial maps (Figure 3) document dynamic cellular reorganization following injury, including the emergence and resolution of populations such as reaEGC, obNBL, and MCG that appear specifically post-injury and resolve by 30 DPI. This temporal progression parallels microglial responses in mammalian traumatic brain injury, where early microglial activation can support neuronal survival and tissue repair through cytokine and chemokine signaling (Obukohwo et al., 2024), whereas prolonged activation contributes to chronic neuroinflammation and secondary neurotoxicity, pointing to shared injury-response dynamics despite divergent regenerative outcomes. The 180 hub genes span all regeneration stages and multiple cell-types, with complete annotations including cell-type assignments, module membership, connectivity metrics, and hub frequencies provided in Supplementary Data Sheet 5. This atlas enables researchers to prioritize candidates based on hub frequency, evolutionary conservation, spatial distribution, or functional annotation, facilitating the transition from descriptive transcriptomics toward hypothesis-driven experimental investigation.

This computational approach complements recent experimental studies in axolotl regeneration by providing network-level context for gene interactions. While STRING analysis using *X. laevis* introduces uncertainty for lineage-specific genes, it enables identification of conserved regulatory modules that may guide cross-species comparisons. The integration of spatial transcriptomics with co-expression networks offers a systems-level perspective that individual gene lists cannot capture.

The spatiotemporal patterns identified here provide starting points for experimental investigation. Future studies could test whether persistent TRH-MSN co-expression reflects active signaling or an incidental injury response, examine the contribution of transiently expressed genes like those in microglia to early tissue reorganization, and assess whether hub genes with conserved regenerative roles actively participate in axolotl brain repair or simply mark cellular state transitions. Targeted perturbation of individual candidates or coordinated gene modules would distinguish drivers of regeneration from correlated transcriptional responses.

This spatiotemporal atlas of hub gene expression provides a framework for prioritizing candidates in axolotl brain regeneration. While computational findings require experimental validation, the integration of network analysis with spatial transcriptomics reveals organizational principles that may guide mechanistic studies in regenerative neuroscience.

## 5 Conclusion

In this study, we provide a descriptive overview of gene co-expression dynamics during axolotl telencephalon regeneration by integrating hdWGCNA with spatial transcriptomics and network-based contextualization through STRING. This approach allowed us to identify hub genes and characterize their spatiotemporal patterns across the regeneration timeline, highlighting both broadly conserved responses and stage-specific transcriptional states. Among these, TRH emerged as the most consistently detected hub across time points, displaying a stable spatial association with MSN-enriched regions; however, this pattern should be interpreted as a regeneration-associated transcriptional state rather than a functional signature. Other hub genes exhibited more restricted temporal or cellular distributions, contributing to a heterogeneous landscape of transcriptional configurations engaged after injury.

Because functional information remains limited for many axolotl genes, our interpretation focused on candidates supported by available empirical or bibliographic evidence, ensuring that conclusions remain grounded in current knowledge while avoiding speculative assignments of biological roles. Overall, this integrative strategy offers a foundational map of regeneration-associated transcriptional organization and provides a framework for prioritizing genes for future mechanistic and experimental studies aimed at elucidating the molecular basis of axolotl brain repair.

## Data Availability

The original contributions presented in the study are included in the article/Supplementary Material, further inquiries can be directed to the corresponding author.
