# Supplementary material for: High-dimensional co-expression network analysis reveals persistent TRH gene expression throughout axolotl telencephalon regeneration
Source: Front Bioinform. 2026 Jan 12;5:1697212. doi: 10.3389/fbinf.2025.1697212 (PMC12832632; doi:10.3389/fbinf.2025.1697212)
Supplement: Supplementary file 1 [file Table1.pdf]

SUPLEMENTARY TABLE 1

Gene	Cell Type	DPI	Function	Species	Reference
MMP2 (metaloproteinasa de matriz 2)	reaEGC, mpEX	2DPI	Formation of blood vessels, wound healing; Axonal regeneration; damage response; Degrades the extracellular matrix; Development of metastases; TGF-β signalling pathway; Responsible of collagen degradation	Mouse, Human, Zebrafish, Axolotl	[1–5]
KTR19 (keratin-19)	reaEGC	2DPI, 15DPI	Intermediate filament cytoskeleton forming; Dedifferentiation of cancer cells; Marks long-lived and resistant cells; Associated with injury response and restoration; NOTCH signaling pathway	Mouse, Human,	[6–10]
KRT18 (keratin-18)	reaEGC	2DPI	Regulation of cell viability, invasion, migration and adhesion; Modulates Alternative Splicing of Genes Involved in Proliferation and Apoptosis; Retains cells in immature stages.	Human,	[11–14]
TNC (Tenascin-C)	reaEGC	2DPI	Related to extracellular matrix formation, migration, proliferation and stemness; Its abundance in the extracellular matrix promotes neural stem and progenitor cells; Facilitates the formation of neuroimmune synapses.	Mouse, Human, Axolotl, Zebrafish,	[15–19]
IQCA1	reaEGC	2DPI	Possible representation as a cell dissemination pathway	Human, Fish	[20]

(IQ Motif Containing With AAA Domain 1)					
RPRML (reprimo-like)	reaEGC, sstIN	5DPI, 30DPI	Associated with embryogenesis, it plays an important role in the production, formation and development of blood cells from haematopoietic stem cells (HSCs) during late fetal and postnatal stages. Conserved expression pattern human - zebrafish.	Human, Zebrafish,	[21,22]
N4BP3 (NEDD4-binding protein 3)	reaEGC	10DPI	Related to the formation of new blood vessels, axonal branching and early neural development; Associated with regulation of innate immune response and metastasis in cancer.	Mouse, Human	[23,24]
COL1A2 (Collagen Type I Alpha 2 Chain)	CP, reaEG	10DPI, 15DPI	Promotes cancer cell proliferation; Involve in development muscle process; Impact on cancer cell migration	Mouse, Human, Axolotl, Zebrafish	[25–28]
LOC112116122	reaEGC, sstIN	15DPI			
WDR45B (WD Repeat Domain 45B)	reaEGC, npyIN	15DPI, 60DPI	Maintains neuronal autophagy and cognitive function; Regulate autophagosome maturation into autolysosomes in neural cells	Mice, Human	[29,30]
POLR2F(DNA-directed RNA polymerase II subunit F)	reaEGC	15DPI	Importance in carcinogenesis and targeted cancer therapy and autoimmune diseases; its expression is related to an attempt to counteract the stress of oxygen deprivation (hypoxia) or to	Human	[31–33]

			protect cells against the damaging effects of ageing.		
DHTKD1 (dehydrogenase E1 and transketolase domain containing 1)	MCG	2DPI	Related to cell proliferation during mouse liver regeneration and mitochondrial energy regulation; associated with peripheral neuropathies/neuromuscular disorders; Linked to impaired insulin sensitivity, cardiovascular disease risks, and Charcot-Marie-Tooth neuropathy.	Mouse, Human	[34–36]
TRH (Thyrotropin-Releasing Hormone)	MCG, MSN, obNBL, scgIN, tlNBL, CP, IMN	5DPI, 10DPI, 20DPI, 30DPI, 60DPI	Neuroprotective effects in experimental brain or spinal cord and other physiological actions (autonomic, analeptic, and endocrine) undesirable for the treatment of neurotrauma; Protect neurons from ischemia and various cytotoxins; Highly conserved in vertebrates.	Human, Axolotl, tiger salamander, frog	
AIMP2 (Aminoacyl TRNA Synthetase Complex Interacting Multifunctional Protein 2)	MCG	5DPI	Released into the extracellular space; Target in cancer; Related to proliferation, migration and invasion of cancer cells; Essential morphological indicators of peripheral nerve degeneration; Marker of glial inflammation in the ventral midbrain.	Mouse, Human	[37–40]
			Se ha observado que sus niveles de expresiòn se correlacionan entre células como regulador.		

ZNF365 (Zinc finger protein 365)	MCG	15DPI	Exhibits protective functions by preventing the senescence of damaged cells; Acts as regulator of neurogenesis and negatively controls neurite outgrowth; Defends zebrafish embryos against gram-negative bacterial infections.	Human, Mice, Zebrafish	[41–44]
ANAPC11 (Anaphase Promoting Complex Subunit 11)	MCG	10DPI	Promotes increased proliferation and invasiveness of cells; Participate in cell cycle regulation	Human, Mice	[45,46]
MAFK (MAF BZIP Transcription Factor K)	MCG	20DPI	Participate in transcriptional activation or represison; Promoted cell proliferation; Linked to the Wnt signaling pathway; Acts as a regulator of key genes related to cellular senescence and ageing.	Human, Mice, Zebrafish	[47–49]
RPL8 (Ribosomal Protein L8)	MCG	20DPI	Play an essential role in cancer prognosis, and significantly correlate with ferroptosis-related genes; Positively correlated with liver regeneration and immune system process; Involved in protein synthesis processes; Associated with age	Human, Mouse, Zebrafish	[50–52]
SCGN (Secretagogin)	obNBL, scgnIN, MSN,npyIN	2DPI, 10DPI, 15DPI, 60DPI	Synaptic transmission regulator; Involved in β-cell maintenance, neuronal functions and efficacy of insulin; Tightly associated with age; Regulates the secretion of hormones and	Human, Mouse, Zebrafish	[53–59]

			neurotransmitters; Is a marker of neuroblasts commuting.		
PCP4L1 (Purkinje Cell Protein 4 Like 1)	obNBL	5DPI	Mainly expressed in the structures of the circumventricular organs, including telencephalic choroid plexi of developing brain; Increase β-Cell proliferation; Involved in the organisation and regulation of early brain development and in the maintenance of specific functions in adult brain structures.	Human, Mice	[60,61]
BTG1 (Bcell translocation gene 1)	obNBL	5DPI	Play a key role in proliferation, apoptosis, and cell growth via modulating transcription, post-transcriptional, and translation; Regulates quiescence in haematopoietic lineages; Controls proliferation in adult stem/progenitor cells of dentate gyrus and SVZ	Mice, Human, Hydra vulgaris, Zebrafish, Xenopus laevis,	[62–67]
THSD4 (Thrombospondin Type 1 Domain Containing 4)	obNBL	5DPI	Play an important role in the extracellular matrix (ECM) during the perinatal transition; Play key roles in tumorigenesis and development of cancer involved in cell cycle and Endocytosis; Involved in the drug resistance and tissue development.	Human, Mice	[68–71]
TIMM13 (translocase of inner mitochondrial membrane 13)	obNBL	5DPI	Vital for the integrity and function of mitochondria; It overexpression increased ATP contents, enhancing cancer cell proliferation and migration; Modulate the tumor immune	Human, Mice	[72–74]

			microenvironment and lead to a poorer prognosis; Essential for the import of proteins into the mitochondrial inner membrane.		
TMEM163 (Transmembrane Protein 163)	obNBL	20DPI	Have a role in myelin development and is associated with hypomyelination leukodystrophy; promote substance exchange and energy supply; Associated with Alzheimer's disease in various tissues.	Human, Mice, Zebrafish	[75–77]
FBXL17 (F-box/LRR-repeat protein 17)	obNBL	20DPI	Accesses a degradation signal at the BTB dimer interface in nonphysiological, nonfunctional complexes; Biomarker for breast cancer therapy; Play a role in coronary heart disease; Associated with attention-deficit/hyperactivity disorder.	Human, Mice, Xenopus	[78–81]
SRGAP1 (SLIT-ROBO Rho GTPase Activating Protein 1)	obNBL	20DPI	Regulator of a proliferative-to-invasive switch in cancer cells; Axonal enhancers in the injured group; Mediates diverse cellular processes such as proliferation, neurogenesis and axon guidance; Biomarker and therapeutic target candidate due to it implication in neuronal functions.	Humna, Mice, Zebrafish	[82–84]
OSBP (Oxysterol-binding protein)	obNBL	20DPI	Present at membrane contact sites and act as either lipid transporters or sensors that control lipid metabolism, cell signaling, and vesicle transport; Implicated in regulated neurite outgrowth and elongation.	Human, Mice, C. Elegans	[85,86]

CARTPT (cocaine and amphetamine-regulated transcript)	MSN, scgnIN, WSN, nptxEX	5DPI, 10DPI, 20DPI, 30DPI, 60DPI	Neuropeptide conserved among multiple mammalian species; Involved in reward, reinforcement, and stress responses - Its strongly correlated with vulnerability to anxiety-like behavior; Key molecule involved in dopamine metabolism;	Human, Mice, Zebrafish	[87–91]
LOC115478996	WSN	5DPI			
CIRBP (Cold-inducible RNA-binding protein)	IMN	15DPI	Plays a cytoprotective role during limb regeneration; Attenuates hypoxia induced insufficient energy production and oxidative stress; May license BrM invasion and recurrence and may foster invasion within the brain.	Human, Mice, Zebrafish, Xenopus l., Axolotl	[92,93]
RUBCN (Rubicon Autophagy Regulator)	IMN	10DPI	Is a negative regulator of autophagy and increases in aged worm tissues at transcript and/or protein levels; Rubicon is required for an age-dependent increase of exosome release.	Human, Mice, Zebrafish, Fly	[94]
BRD2 (Bromodomain-containing protein 2)	IMN	10DPI	Is Needed for Proper Circulatory, Excretory and Central Nervous System Formation and Act as Genetic Antagonists during Development; Affects aging by protecting against the accumulation of molecular and cellular damage.	Human, Mice, Zebrafish	[95,96]
MRPL52 (Mitochondrial	IMN	15DPI	Mediates hypoxia-induced apoptotic resistance and	Human, Mice	[97]

Ribosomal Protein L52)			metastatic initiation of breast cancer.		
GTPBP6 (GTP Binding Protein 6)	IMN	15DPI	Has a dual function acting in ribosome recycling and biogénesis;	Human, Mice	98
PTAR1 (prenyltransferase alpha subunit repeat containing 1)	IMN	15DPI	Involve in inflammatory response as a pre-requisite for innate immunity in cells.	Human	99
NBN (Nibrin)	IMN	15DPI	Apoptosis markers; Induces proliferation and enhanced apoptosis of cells;	Human, Mice	100
ANAPC15 (Anaphase Promoting Complex Subunit 15)	IMN, rIPC1, mpIN	5DPI, 15DPI	Involved in regulation of mitotic cell cycle spindle assembly checkpoint; Part of anaphase-promoting complex.	Human,	101
CRIP1 (cysteine-rich intestinal protein)	rIPC1	15DPI	Expression changes in response to cellular damage due to injury, with a functional role in proliferation, differentiation, or turnover; Play an important role during embryonic development.	Human, Mice, Zebrafish, Murine, Xenopus laevis,	102–104
SELENOH (Selenoprotein H)	rIPC1	15DPI	It functions as an oxidoreductase, and has been shown to protect neurons against UVB-induced damage by inhibiting apoptotic cell death pathways, promote mitochondrial biogenesis and mitochondrial function, and suppress cellular senescence through genome maintenance and redox regulation; Decreased	Human, Zebrafish	105

			DNA damage and oxidative stress.		
FPGS (Folylpolyglutamate Synthase)	nptxEX, sfrpEGC	2DPI, 60DPI	Is essential for folate homeostasis and the survival of proliferating cells; Its expression significantly impacts plasticity and cell fate.	Human, Mice, Zebrafish	[106,107]
ENKD1 (Enkurin Domain Containing 1)	nptxEX	2DPI	Regulates the proliferation, migration and invasion of cancer cells; Critical function in regulating cellular homeostasis;	Human, Mice,	[108–110]
SRRT (Serrate)	nptxEX	5DPI	Regulate cell cycle, survival, and differentiation	Human, Mice	[111]
BTBD2 (BTB Domain Containing 2)	nptxEX	10DPI	Involved in the double-strand break repair pathway; Relate with immune-inflammatory.	Human, Mice	[112]
NMB (Neuromedin B)	nptxEX, obNBL, dpEX, sfrpEGC	5DPI, 15DPI, 30DPI	Initiates the reprograming of Schwann cells, which then direct axon regeneration; Plays a pivotal role in the transmission of neuroinflammation	Human, Mice, Xenopus laevis,	[113–115]
ZNRD1 (zinc ribbon domain-containing 1-antisense 1)	nptxEX	15DPI	Involve in cell proliferation, migration, and angiogenesis; associate with post-traumatic stress disorder	Human, Mice	[116,117]
UBE3B (Ubiquitin Protein Ligase E3B)	nptxEX	15DPI 1	Involved in human hippocampal neuron injury; Mediated cancer growth and lung metastasis	Human, Mice,	[118,119]
DLG5 (Discs Large MAGUK	nptxEX	20DPI	Plays a role in cell migration, cell adhesion, precursor cell division,	Human, Mice,	[120,121]

Scaffold Protein 5)			cell proliferation, epithelial cell polarity maintenance, transmission of extracellular signals to the membrane and cytoskeleton, inflammatory bowel disease and Crohn's disease; DLG5 is essential for cilia.	Zebrafish, Xenopus laevis	
MRPL17 (Mitochondrial Ribosomal Protein L17)	nptxEX	30DPI	Associated with neuropathology and cancer biomarker; expressed throughout early embryogenesis with little stage or tissue specificity.	Human, Mice	[122–124]
MEGF11 (Multiple EGF Like Domains 11)	dpEX, wntEGC, npyIN	10DPI, 15DPI, 60DPI	Plays a key role in tumour survival and induces a cytokine and a chemokine cascade; promotes proper Purkinje cell development and cerebellar network formation by regulating immature synaptic transmisión.	Human, Mice	[125,126]
ZADH2 (zinc binding alcohol dehydrogenase domain containing 2)	dpEX	10DPI	Involve in cell proliferation and apoptosis; Correlated with the decline of renal function.	Human, Mice	[127,128]
STAT1 (Signal Transducer And Activator Of Transcription 1)	dpEX	15DPI	Promote axon regeneration after injury; Involve in angiogenesis, and blood flow recovery; Widely expressed throughout the developing embryo.	Human, Mice, Zebrafish, Xenopus laevis, Golden-line barbel fish	[129–131]

PDK3 (pyruvate dehydrogenase kinases)	dpEX	30DPI	Important in cell dedifferentiation and proliferation after injury; Glycolysis checkpoint protein for Hematopoietic stem cells quiescenc	Human, Mice, Zebrafish, C. elegans	[132–135]
C1QL1 (Complement C1q Like 1)	mpEX, sstIN	5DPI, 15DPI , 60 DPI	Involve in the cell differentiation and myelin production during development; Stimulate the new blood vessel growth; Is conserved across vertebrate species	Human, Mice, Zebrafish	[136,137]
RGS2 (Regulator Of G Protein Signaling 2)	mpEX	10DPI	Key molecular regulator in neural crest development; modulator of neuronal toxicity; Involve in hippocampal neuron regeneration and rescues depression-like behavioral impairments; Promote anti-inflammatory function in lung injury.	Human, Mice, Zebrafish, Xenopus laevis	[138–141]
KCNG3 (Potassium Voltage-Gated Channel Modifier Subfamily G Member 3)	mpEX	30DPI	Present role for these genes in colonic motility; Involve in the electrical functioning of interstitial cells of the mouse colon.	Human, Mice	[142]
CCT3 (chaperonin-containing TCP-1 subunit 3)	tlNBL, wntEGC	2DPI 5DPI	Implicated in several types of malignant tumors' development; Relate to cell proliferation, cell cycle progression and has induced cell apoptosis in vitro; Plays an essential role in retinotectal development.	Human, Mice, Zebrafish	[143,144]

BUD31 (BUD31 Spliceosome Associated Protein)	tlNBL, ribEGC	2DPI, 30DPI	Regulates the self-renewal and differentiation of male germ cells in mammals; Increased processes related to cancer cell migration and proliferation.	Human, Mice, Xenopus laevis, C. elegans	[145,146]
RECQL (RecQ Like Helicase)	tlNBL,	2DPI	Involve in female meiosis/germ cell differentiation and spermatogenesis; Plays a critical role in sustaining DNA synthesis under conditions of replication stress; Represent a target for cancer therapy.	Human, Mice	[147–149]
PCGF6 (Polycomb Group Ring Finger 6)	tlNBL	2DPI	Nonredundantly involved in maintaining the pluripotent nature of cells and it functions in induced pluripotent stem reprogramming; Regulate germ cell-related genes.	Human, Mice, Zebrafish, Murine	[150–152]
PICK1 (Protein Interacting With PRKCA 1)	tlNBL	2DPI	Constitutes a valid drug target for treating inflammatory and neuropathic pain conditions; Is localized at the perinuclear region as well as specialized structures such as synapses of neurons.	Human, Mice, C. elegans, Xenopus l.	[153,154]
SPART (Spartin)	tlNBL	2DPI	Receptor for autophagy of lipid droplets that plays an important role in the turnover of triglycerides in motor neurons.	Human, Mice	[155]
CHST8 (Carbohydrate Sulfotransferase 8)	tlNBL	5DPI	Breast cancer cells expressing CHST8 suppress T cell activation, and Chst8 loss attenuates tumor growth in a syngeneic mouse model.	Human, Mouse	[156]

EHBP1 (EH Domain Binding Protein 1)	tlNBL	5DPI	Identified to be closely related to the repair process in oocytes; Conserved regulator of endocytic recycling; switch that dictates the direction of Wg/Wnt polarized intracellular transport	Human, Mice	[157–159]
TBC1D14 (TBC1 Domain Family Member 14)	tlNBL	60DPI	Inhibited the migration and invasion of carcinoma cells in vivo and induced autophagy; regulates autophagy via the TRAPP complex and ATG9 traffic in mammalian cells.	Human, Mice	[160,161]
INPP1 (inositol polyphosphate 1-phosphatase)	tlNBL	5DPI	Is highly expressed in aggressive human cancer cells and primary high-grade human tumors; Involve in cancer cell motility, invasiveness, and tumorigenicity; Associated with autistic disorder.	Human, Mice	[162,163]
GAR1 (GAR1 Ribonucleoprotein)	tlNBL	15DPI	Contribute to sub-nucleolar phase separation; novel interactors of spinal muscular atrophy (SMN) and suggest a function for the SMN complex; Involve in cell proliferation and the activation of DNA damage.	Human, Mice, Zebrafish, C. elegans, Xenopus l.	[164–166]
USP25 (Ubiquitin Specific Peptidase 25)	tlNBL	15DPI	Involve in zebrafish development; attenuates liver injury and reduces the mortality rates resulted; Inhibits Neuroinflammatory Responses After Cerebral Ischemic Stroke	Human, Mice, Zebrafish	[167–169]

CFAP20 (Cilia And Flagella Associated Protein 20)	SfrpEGC, MCG	2DPI, 10DPI	In zebrafish, cfap20 is required for motile cilia function, and in C. elegans, CFAP-20 maintains the structural integrity of non-motile cilia inner junctions, influencing sensory-dependent signalling and development.	Human, Mice, Zebrafish, C. elegans	[170]
BCAS3 (BCAS3 Microtubule Associated Cell Migration Factor)	sfrpEGC	2DPI	Critical in angiogenesis and implicated in human embryogenesis and tumorigenesis; Vital role in neural tissue development.	Human, Mice, Zebrafish, C. elegans	[171]
RBL2 (RB Transcriptional Corepressor Like 2)	sfrpEGC	2DPI	Cell cycle regulator; Involve in mitochondrial damage, apoptosis in vitro and differentiation of human embryonic stem cells.	Human, Mice, Zebrafish, Xenopus tropicalis	[172,173]
EGF (Epidermal Growth Factor)	sfrpEGC, npyIN	5DPI, 10DPI	Common feature of blastemas capable of regenerating limbs and fins in a variety of highly regenerative species; Promotes progenitor cell proliferation and sensory neuron regeneration in the zebrafish olfactory epithelium; Involve in developmental human placenta	Human, Mice, Axolotl, Lungfish, Polpyterus, Xenopus laevis, Zebrafish	[174,175]
CH25H (cholesterol 25-hydroxylase)	sfrpEGC	5DPI	Protects the cells; Involve in prevention of proinflammatory state of lipid-droplet-accumulating microglia/astrocytes to limit excessive inflammation; Exhibited increased phagocytosis	Human, Mice, Zebrafish	[176–178]

			and neuroprotective property after stroke		
KDM5C (Lysine Demethylase 5C)	sfrpEGC	60DPI	Associated with embryonic development and provided additional information regarding the complex and dynamic gene network that regulates neural crest formation and eye development; KDM5C is identified as a safeguard to ensure that neurodevelopment occurs at an appropriate timescale.	Human, Mice, Xenopus, Zebrafish	[179,180]
MICOS13 (Mitochondrial Contact Site And Cristae Organizing System Subunit 13)	sfrpEGC, sstIN	10DPI, 15DPI	Plays crucial roles in the maintenance of cristae junctions at the mitochondrial inner membrane; Cause hepato-encephalopathy.	Human	[181]
PDP1 (Pyruvate Dehydrogenase Phosphatase Catalytic Subunit 1)	sfrpEGC	10DPI	Highly expressed in the cerebral cortex, hippocampus and thalamus of rat; Promotes the progression of cancer	Human, Mice	[182]
UBAC1 (UBA Domain Containing 1)	sfrpEGC	15DPI	Participates in the inflammatory signal transduction pathways	Human, Mice	[183]
JAM2	sfrpEGC	15DPI	Involve neurovascular unit disruption; Is a necessary potentiator of the disomic genetic modifier; In vascular junctions may play a role in regulating	Human, Mice, Xenopus l.	[184]

(Junctional Adhesion Molecule 2)			vascular function in vivo; Regulate cell cycle progression and cell adhesión.		
CAV1<br><br>(Caveolin 1)	sfrpEGC	15DPI	Play an anti-fibrotic effect in vitro and in vivo; Protects retinal neurons against excitotoxicity; Neuroprotective effect	Human, Mice, Zebrafish, Salamander,Xenopus l.	[185–187]
C1QTNF4<br><br>(C1q And TNF Related 4)	sfrpEGC	15DPI	Is involved in the regulation of the inflammatory pathways by pro-inflammatory function; Has been linked to the autoimmune disease systemic lupus erythematosus through genetic studies; In human induced pluripotent stem cell-derived neurons.	Human, Mice, Zebrafish	[185,188,189]
WNT7B<br><br>(Wnt Family Member 7B)	cckIN, sfrpEGC	5DPI, 20DPI	Have a role in the patterning and morphogenesis of forebrain regions; Is expressed during early brain development; Involve in alveolar regeneration and repair of injury/fracture.	Human, Mice, Zebrafish, Xenopus laevis	[190–194]
USP8<br><br>(Ubiquitin Specific Peptidase 8)	SfrpEGC	20DPI	Regulates hepatocellular carcinoma tumorigenesis and confers ferroptosis resistance; Involve in a critical signaling cascade that restricts ciliogenesis in dividing cells, and functions to facilitate cell proliferation.	Human, Mice, Zebrafish, Xenopus laevis	[195,196]
RASSF8	SfrpEGC	20DPI	Regulated autophagy, proliferation and proapoptotic	Human, Mice,	[197,198]

(Ras Association Domain Family Member 8)			effects in muscle cells; Associated with vascular endothelial growth factor; Expressed throughout the murine embryo and in normal human adult tissues; Define as a tumor suppressor gene that is essential for maintaining adherens junction function in epithelial cells and have a role in epithelial cell migration.	Xenopus, Zebrafish,	
TEX264 (Testis Expressed 264)	SfrpEGC	20DPI	Drives selective autophagy of DNA lesions to promote DNA repair and cell survival; Is a receptor for autophagic degradation of the endoplasmic reticulum	Huma, Mice, Zebrafish	[199,200]
MEGF10 (Multiple EGF-like domains 10)	sfrpEGC, wntEGC	30DPI , 60DPI	A key role in human skeletal muscle and suggest satellite cell dysfunction as a novel myopathic mechanism; Regulate skeletal muscle stem cell migration and muscle regeneration.	Human, Mice, Zebrafish	[201,202]
PLXNB1 (Plexin B1)	SfrpEGC	30DPI	Involve in signal transduction pathway controlling cell repulsión fundamental to multiple developmental processes; Involve in tumor growth and metastatic.	Human, Mice, Xenopus l.	[203,204]
ANKS1A (Ankyrin Repeat And Sterile Alpha Motif Domain Containing 1[a])	SfrpEGC	30DPI	Regulates cerebrovascular clearance in brain endothelial cells; Regulate Cancer Cells Motility; Essential for proper development of ependymal cells	Human, Mice, Xenopus l.	[205–207]

LRRC40 (Leucine Rich Repeat Containing 40)	wntEGC, SfrpEGC	30DPI, 60DPI	Candidate genes in autism spectrum disorder; Involve in TNFR and TLR signaling that showed an immediate/early pulse regulation in Hydra regenerative contexts;	Human, Hydra vulgaris	[64]
GJA1 (Gap Junction Protein Alpha 1)	SfrpEGC	60DPI	Required in the population of dividing cells during fin regeneration; Regulate cell proliferation and segment length; Controls proliferation and apoptosis in human; Relate to cell communication in embryo Development.	Human, Mice, Zebrafish, Xenopus l.	[208,209]
GDI2 (GDP Dissociation Inhibitor 2)	SfrpEGC	60DPI	Regulator of Rab GTPase activity and intracellular vesicle and membrane trafficking; Associated with cognitive impairment and neuronal loss	Human, Mouse, Amphibia	[210]
GATD1 (Glutamine Amidotransferase Class 1 Domain Containing 1)	SfrpEGC	60DPI	No realate information found		
KDM5C (Lysine Demethylase 5C)	SfrpEGC	60DPI	Controls WNT output to regulate the timely transition of primary to intermediate progenitor cells and consequently neurogénesis; Crucial sentinel for neurodevelopment; Associated with embryonic development, neural crest formation and eye development.	Human, Mice, Zebrafish, Xenopus laevis	[179]

COMMD7 (COMM Domain Containing 7)	SfrpEGC	60DPI	Regulator of Zebrafish thrombus formation; Plays an important role in the late progression of cancer; Involve in hepatocellular carcinoma growth.	Human, Mice, Zebrafish	[211,212]
SAR1B (Secretion Associated Ras Related GTPase 1B)	wntEGC	5DPI	Highly conserved among vertebrates; broadly expressed during development and enriched in the digestive tract organs, brain, and craniofacial skeleton; Required for growth of exocrine pancreas and liver; promotes mTORC1-dependent growth of lung tumours in mice.	Human, Mice, Zebrafish	[213,214]
ERI3 (ERI1 Exoribonuclease Family Member 3)	wntEGC	10DPI	Play a key role in assessing the prognostic risk of ischaemic stroke; was found to be associated with schizophrenia.	Human, Mice	[215,216]
RABGGTA (Rab Geranylgeranyltransferase Subunit Alpha)	wntEGC	10DPI	Implicated in the Hermansky-Pudlak syndrome (HPS) genes associated with HPS in pinky; Is a candidate gene for hair color.	Human, Mice	[217]
C1QB (Complement C1q B Chain)	wntEGC	15DPI	Is involved in the classical pathway and are conserved functionally from fish to human; Had the potential to serve as prognostic or predictive markers for neuropathic pain	Human, Mice, Zebrafish	[218,219]
SDF2L1	wntEGC	15DPI	Inhibits Cell Proliferation, Migration, and Invasion in cancer; Controls feeding-induced	Human, Mice	[220,221]

(Stromal Cell Derived Factor 2 Like 1)			ER stress and regulates metabolism.		
STRN4<br><br>(Striatin 4)	wntEGC	15DPI	Associated with tumor progression; Regulate proliferation, migration, invasión, metastasis and the anchorage-independent growth of cancer cells.	Human, Mice	[222]
CFAP221<br><br>(Cilia And Flagella Associated Protein 221)	wntEGC	20DPI	Involve in ciliary motility and assembly of the central pair apparatus in mice.	Human, Mice	[223]
ST6GALNAC2<br><br>(ST6 N-Acetylgalactosaminide Alpha-2,6-Sialyltransferase 2)	wntEGC	30DPI, 60DPI	Acts as a breast cancer metastasis suppressor	Human, Mice	[224]
MORF4L1<br><br>(Mortality Factor 4 Like 1)	wntEGC	60DPI	Promote the proliferation of injured tenocytes in vitro in tendon injury.	Human, Mice	[225]
FAM43A<br><br>(Family With Sequence Similarity 43 Member A)	sstiIN	2DPI	Diseases associated with FAM43A include Chromosome 3Q29 Microdeletion Syndrome and Cholestasis-Lymphedema Syndrome.	Human, Mice	NA

RBM7 (RNA Binding Motif Protein 7)	sstIN	2DPI	Related to defects in motor neurons and cerebellum; Involved in constellations of pontocerebellar hypoplasia (PCH), spinal muscular atrophy (SMA), and central nervous system demyelination; is up-regulated in the injured lung epithelium and disturbs normal epithelial cell repair and regeneration by promoting apoptosis of damaged epithelial cells; stimulates RNA polymerase II (Pol II) transcription and promotes cell viability	Human, Mice, Zebrafish	[226–229]
FBXO3 (F-Box Protein 3)	sstIN	2DPI	Drives neuroinflammation to aggravate cerebral ischemia/reperfusion injury; Has an important impact on the pathophysiology of the inflammatory process; Provides protection in acute lung injury induced by ischemia-reperfusion	Human, Mice	[230,231]
MREG (Melanoregulin)	sstIN	2DPI	Regulates thyroid cancer cell invasion and proliferation; Regulates microtubule-dependent retrograde melanosome transport through the dynein-dynactin motor complex	Human, Mice	[232,233]
RASGRP2 (RAS Guanyl Releasing Protein 2)	sstIN, CP	5DPI, 10DPI	Have been reported in cells of the blood cell lineage; Suppresses apoptosis cells; Is essential for blood vessel formation during Xenopus development	Human, Mice, Xenopus laevis	[234–236]

CHMP2B (Charged Multivesicular Body Protein 2B)	sstIN	10DPI	Involved in autophagy	Human, Mice, Xenopus laevis	[237]
GTF2E1 (General Transcription Factor IIE Subunit 1)	sstIN	10DPI	Related to immune functions; Expressed in cancer cells.	Human, Zebrafish	[238,239]
CDK12 (Cyclin Dependent Kinase 12)	sstIN	10DPI	Relate to protective effects in renal tubular epithelial cells	Human, Mice, Zebrafish	[240]
ICE1 (Interactor Of Little Elongation Complex ELL Subunit 1)	sstIN	10DPI	Involved in transcriptional control	Human, Mice	[241]
PIP5K1B (Phosphatidylinositol-4-Phosphate 5-Kinase Type 1 Beta)	sstIN	15DPI	Relate to tumor immune microenvironment.	Human, Mice, Zebrafish	[242]
ACADM	sstIN	15DPI	Involved in β-oxidation in the ovarian mitochondria	Human, Mice	[243]

(Acyl-CoA Dehydrogenase Medium Chain)					
NCSTN<br><br>(nicastrin)	sstIN	15DPI	In fish, one role of ncstn is the maintenance of pigmentation homeostasis; One of the roles of NCSTN in humans is the prevention of inflammatory processes in the adnexal structures of the skin	Human, Mice, Zebrafish	[244]
KLHL17<br><br>(Kelch Like Family Member 17)	sstIN	60DPI	Promotes the proliferation and migration of non-small cell lung cancer	Human, Mice	[245]
SLC7A8<br><br>(Solute Carrier Family 7 Member 8)	sstIN	60DPI	A novel regulators of left-right asymmetry development in the small fish medaka; highly expressed in eye, in retinal pigmented epithelium, and in tooth buds at day 16.5 of mouse gestation	Human, Mouse, Zebrafish, Xenopus laevis	
L3MBTL1<br><br>(L3MBTL Histone Methyl-Lysine Binding Protein 1)	sstIN	60DPI	Maintain high levels of expression in neurons of the mature brain; operates in both normal stress response and proteotoxicity-associated neurodegenerative diseases; Is conserved from Caenorhabditis elegans to mammalian neurons.	Human, Mice	[246,247]
NPY<br><br>(neuropeptide Y)	sstIN, npyIN	20DPI, 60DPI	Is abundantly present in the terminal nerve in the axolotl; Modulates olfactory epithelial responses; Involved in appetite regulation; were initially	Human, Mice, Axolotl, Zebrafish,	[248–250]

			expressed in distinct neurons, evolution has resulted in their coexpression in mammalian hypothalamic neurons; involved in inhibition of melanotrope cell activity in Xenopus laevis.		
SORCS1<br><br>(Sortilin Related VPS10 Domain Containing Receptor 1)	mpIN	2DPI	Influences renal disease susceptibility in both rat and human; related sorting receptors expressed in neurons of the arcuate nucleus of the hypothalamus.	Human, Mice	[251,252]
SDR16C5<br><br>(Short Chain Dehydrogenase/ Reductase Family 16C Member 5)	mpIN	2DPI	Necessary for embryonic and adult tissue differentiation, development, and apoptosis, and it also participates in immune response and regulates energy metabolism; Acts as a highly active retinol dehydrogenase (rdhe2) that promotes retinoic acid biosynthesis when expressed in mammalian cells	Human, Mice, Xenopus laevis	[253,254]
DTNB<br><br>(Dystrobrevin Beta)	mpIN	2DPI	Relate to neuronal injury and inflammation, potentially by altering cytoskeleton structure, immune activity disinhibition, synaptic transmission, apoptosis and transport among other functions.	Human, Mice, Zebrafish, Xenopus laevis	[255,256]
PEX7<br><br>(Peroxisomal Biogenesis Factor 7)	mpIN	5DPI	Involve in brain development of Drosphila m.; Key role of peroxisomal functioning in the pathogenesis of the human disorder.	Human, Mice, Drosphila m.	[257]

COX3 (cytochrome c oxidase subunit III)	mpIN	5DPI	Related to mitochondrial biogénesis; Expressed in brain and spinal cord	Human, Mice, Zebrafish	258,259
PHLDA1 (Pleckstrin homology-like domain family A member 1)	mpIN, sstIN	30DPI, 60DPI	Critical determinant in immunological regulation and cell apoptosis; Involve in neuroinflammation during cerebral ischemia/reperfusion injury	Human, Mice	260
TRMT9B (TRNA Methyltransferase 9B)	mpIN	30DPI	Regulator of synapse formation, function in Drosophila and is enriched in the nervous system	Human	261
SPEF1 (Sperm Flagellar 1)	mpIN	60DPI	Detected in mammalian cilia; Binds to the microtubule seam and also crosslinks two parallel microtubules; Enables mammalian ciliary central apparatus formation.	Human, Mice	262
FBXO45 (F-Box Protein 45)	mpIN	60DPI	Target additional synaptic and axonal proteins, relate to overlapping neurodevelopmental defects and neuropathic injury.	Human, Mice, Zebrafish	263,264
SEL1L (SEL1L Adaptor Subunit Of SYVN1 Ubiquitin Ligase)	cckIN	10DPI	Conserved gene associated with the endoplasmic reticulum-associated degradation (ERAD) pathway and involved in mediating the balance between stem cells self-renewal and differentiation of neural progenitors.	Human, Mice, Zebrafish	265

ATP6AP1L<br><br>(ATPase H+ Transporting Accessory Protein 1 Like)	cckIN	10DPI	Regulate cell proliferation, migration, and invasión in cancer cells.	Human, Drosphila m.	266
POLE2<br><br>(DNA polymerase epsilon 2)	CP, cckIN	10DPI, 15DPI	Relate to improve DNA damage repair ability; Involve in proliferation of cancer cells and promoted their ferroptosis	Human, Mice, Xenopus laevis	267,268
MED30<br><br>(Mediator Complex Subunit 30)	cckIN	15DPI	Has pathophysiological roles in the proliferation, migration, and invasion of gastric cancer cells; Promoted the expressions of genes related to epithelial-mesenchymal transition and induced a fibroblast-like morphology.	Human, Mice	269
RUVBL2<br><br>(RuvB Like AAA ATPase 2)	cckIN	20DPI	Suppresses Cardiomyocyte Proliferation During Heart Development and Regeneration	Human, Mice, Zebrafish, Xenopus laevis	270
SAMD9<br><br>(Sterile Alpha Motif Domain Containing 9)	cckIN	20DPI	Correlated with immunological response; play a role in the inflammatory response to tissue injury	Human, Mice	271,272
TM2D3<br><br>(TM2 Domain Containing 3)	cckIN	15DPI	Regulate Notch signaling and neuronal function in Drosophila	Human, Mice, Drosphila m.	273

MRPL51 (Mitochondrial Ribosomal Protein L51)	cckIN	15DPI	Expressed in embryos and brains of fetal/postnatal mice	Human, Mice	[274]
NTNG1 (Anaphase Promoting Complex Subunit 15)	npyIN	10DPI	Are expressed in distinct neuronal subsets in a complementary manner and control axonal projections of amygdala	Human, Mice	[275]
DNAAF5 (Dynein Axonemal Assembly Factor 5)	npyIN	10DPI	Promoted the proliferation of cancer cells.	Human, Mice	[276]
ING3 (Inhibitor Of Growth Family Member 3)	npyIN	10DPI	Appear to play a significant role in apoptosis; Is an essential factor for normal embryonic development and that it plays a fundamental role in prenatal brain formation.	Human, Mus musculus, Xenopus laevis, Zebrafish, Caenorhabditis elegans	[277,278]
TRIM67 (Tripartite Motif Containing 67)	npyIN	15DPI	Evolutionarily conserved; Is differentially enriched in specific brain regions during development and adulthood; Necessary in appropriate brain development and behavior.	Human, Mice, Zebrafish	[279]

RABGAP1L (RAB GTPase Activating Protein 1 Like)	npyIN	15DPI	Important in brain development.	Human, Mice	[280]
NCKIPSD (NCK Interacting Protein With SH3 Domain)	npyIN	60DPI	Involved in the Rho signaling pathway; Identified as anxiety and stroke gene.	Human, Mice	[281]
PRXL2A (Peroxiredoxin Like 2)	npyIN	20DPI	Reported to be an antioxidant protein that protects cells from oxidative stress.	Human, Mice	[282]
MCOLN1 (Mucolipin TRP Cation Channel 1)	scgnIN	15DPI	Have a role in embryonic development, hair cell viability and cellular maintenance.	Human, Mice, Zebrafish, Xenopus l.	[283]
MFN1 (Mitofusin 1)	scgnIN	20DPI	Regulate neural stem cell (NSC) self-renewal; Mediates neural induction in pluripotent stem cells.	Human, Mice	[284]
SYT10 (Synaptotagimin 10)	scgnIN	10DPI	Involvement in exocytosis of secretory vesicles in neurons; An important role in the neuronal response to strong synaptic activity as a consequence of excitotoxic insults.	Human, Mice, Zebrafish	[285,286]

TBC1D10A (TBC1 Domain Family Member 10A)	scgnIN	20DPI	regulate exosome secretion in a catalytic activity-dependent manner.	Human, Mice, Zebrafish	[287]
MNT (MAX Network Transcriptional Repressor)	scgnIN	30DPI	Associated with cell proliferation, apoptosis and cell cycle.	Human, Mice, Zebrafish, Xenopus laevis, Pleurodeles waltl	[288]
YES1 (YES Proto-Oncogene 1)	scgnIN	60DPI	Promising target for suppression of metastasis.	Human, Mice, Zebrafish	[289]
PIM1 (Pim-1 Proto-Oncogene, Serine/Threonine Kinase)	scgnIN	60DPI	Promote cancer development.; Plays an important role in erythroid differentiation under hypoxia; Expresión required for normal visual function in Zebrafish.	Human, Mice, Zebrafish, Xenopus laevis	[290–292]
KCNF1 (Potassium Voltage-Gated Channel Modifier Subfamily F Member 1)	scgnIN	15DPI	Regulate cell proliferation, tumor progression and promotes lung cancer.	Human, Mice	[293]
CHMP3	CMPN	5DPI	Linked to extracellular interactions, suggesting its involvement in cellular communication and tissue repair	Human, Mice	[294]

(Charged Multivesicular Body Protein 3)			mechanisms essential for wound healing.		
CBL (Cbl Proto-Oncogene)	CMPN	5DPI	Modulate Xenopus development	Human, Mice, Zebrafish, Xenopus laevis	[295]
ZNF385D (Zinc Finger Protein 385D)	CMPN, scgnIN	10DPI, 20DPI	Linked to psychiatric stress disorders	Human, Mice	[296]
PGAM2 (Phosphoglycerate Mutase 2)	CMPN	10DPI, 15DPI	Plays a pivotal role in glycerol degradation, thereby facilitating the proliferation and differentiation of satellite cells in skeletal muscle	Human, Mice	[297]
CACNA1G (Calcium Voltage-Gated Channel Subunit Alpha1 G)	CMPN	15DPI	High expressed in the thalamus and is further increased in thalamic neurons treated	Human, Mice	[298]
GRK5 (G Protein-Coupled Receptor Kinase 5)	CMPN	20DPI	Important mediator of cardiovascular homeostasis; Control cardiac function as well as morphogenesis during development; Expressed in rat brain; Closely related to cerebral nerve function and neurodegenerative diseases.	Human, Mice, Zebrafish, Xenopus laevis	[299,300]

TNR (Tenascin-R)	CMPN	60DPI	Play important roles in cell proliferation and migration, fate determination, axonal pathfinding, myelination, and synaptic plasticity.	Human, Mice	[301]
RBP4 (Retinol Binding Protein 4)	VLMC	2DPI, 10DPI, 15DPI, 60DPI	Related to cell survival; Can promote the proliferation and migration of cells;	Human, Mice, Zebrafish	[302,303]
ZDHHC20 (Zinc Finger DHHC-Type Palmitoyltransferase 20)	VLMC	2DPI	Affects the interaction of tumor cells and the innate immune system; Promotes metastasis of cancer; Related to regulation of cell proliferation and apoptosis.	Human, Mice	[304,305]
SMOC2 (SPARC Related Modular Calcium Binding 2)	VLMC	20DPI	Is expressed in many different tissues and was shown to enhance the response to angiogenic growth factors, mediate cell adhesion, keratinocyte migration, and metastasis; Modulates embryonic myelopoiesis during zebrafish development	Human, Mice, Zebrafish	[306]
COL18A1 (Collagen Type XVIII Alpha 1 Chain)	VLMC	15DPI	Gen associated with structural brain anomalies	Human, Mice, Zebrafish	[307]
KCNB2	CP, MSN	10DPI, 30DPI	Involved in neurodevelopmental disorders	Human, Mice	[308]

(Potassium Voltage-Gated Channel Subfamily B Member 2)		, 60DPI			
SARNP (SAP Domain Containing Ribonucleoprotein)	CP	10DPI	Play a role in cell cycle progression.	Human, Mice	309
ZCCHC2 (Zinc Finger CCHC-Type Containing 2)	CP	10DPI	Promoted cell proliferation and plays a role in the regulation of RB tumorigenesis	Human	310
SUCLG2 (Succinate-CoA Ligase GDP-Forming Subunit Beta)	CP	10DPI	Mutations lead to a mitochondrial disorder manifesting as encephalomyopathy with dystonia, deafness and lesions in the basal ganglia	Human, Mice, Zebrafish	311
RBM11 (RNA Binding Motif Protein 11)	CP	10DPI	Splicing factor with potential implication in the regulation of alternative splicing during neuron and germ cell differentiation	Human, Mice	312
PIP5K1A (Phosphatidylinositol-4-Phosphate 5-Kinase Type 1 Alpha)	CP	10DPI	Hepatocyte proliferation and liver regeneration after partial hepatectomy are suppressed in Pip5k1a knockout mice	Human, Mice, Zebrafish	313

OXCT1 (3-Oxoacid CoA-Transferase 1)	CP	30DPI	Overexpression of OXCT1 successfully increased hippocampal neurogenesis via activation of Akt/GSK-3β/β-catenin signaling and improved cognitive function	Human, Mice	[314]
ZSCAN2 (Zinc Finger And SCAN Domain Containing 2)	Uknown	15DPI	Could play a role in regulating inflammation and the immune response in the lung that undergoes partial lung regeneration. In Zscan2 knockout mice regeneration after cytotoxic insult was significantly impaired	Human, Mice	[315,316]
PDZRN3 (PDZ Domain Containing Ring Finger 3)	Unknow n	15DPI	Suppresses apoptosis and promotes proliferation in myoblasts. Plays a role during the early phase of muscle regeneration. Regulator of the Wnt pathway which is crucial in Blood brain barrier maintenance.	Human, Mice, Zebrafish, Xenopus laevis	[317,318]
SCYL3 (SCY1 Like Pseudokinase 3)	ntng1IN	15DPI	Regulates neuronal function and survival. Role in maintaining motor neuron viability. Accelerates the onset of the motor neuron disorder caused by Scyl1 deficiency.	Human, Mice	[319]
LOC115636311	ntng1IN	15DPI			
LOC106731590	ntng1IN	15DPI			
DAZAP2	ntng1IN	15DPI	Regulate cell proliferation of undifferentiated human iPSCs	Human, Mice, Zebrafish,	[320]

(DAZ Associated Protein 2)				Xenopus tropicalis	
RANBP2<br><br>(RAN Binding Protein 2)	ntng1IN	20DPI	Play an integral part in the SUMOylation of the Insulin-like Growth Factor 1 Receptor (IGF-1R) and subsequently prevent AD. Play key roles in mediating nuclear migration in proliferating neuronal precursors (RGPs)	Human, Mice, Zebrafish, Xenopus laevis	[321,322]
TNRC6B<br><br>(Trinucleotide Repeat Containing Adaptor 6B)	ntng1IN	20DPI	Involved in micro-RNA-directed RNA processing. Indirect role in gene expression regulation. Associated with neurodevelopmental disorders with or without speech problems	Human, Mice, Xenopus laevis	[323]
ACSBG1<br><br>(Acyl-CoA Synthetase, Bubblegum Family, member 1)	ribEGC	5DPI	Plays a major role in very-long-chain fatty acid metabolism in the brain. ACSBG1 methylation is altered in schizophrenia. May affect synaptic signaling across neuropsychiatric diseases.	Human, Mice	[324]
ERRFI1<br><br>(ERBB Receptor Feedback Inhibitor 1)	ribEGC	5DPI	Significant tumor suppressor gene and is frequently deleted, mutated or downregulated in various types of cancer, including glioblastomas.	Human, Mice	[325]
FZD2<br><br>(Frizzled Class Receptor 2)	ribEGC	5DPI	Involved in Wnt-activated receptor activity. Stimulate the canonical and non-canonical Wnt pathways in malignancies. Meditates the EMT process and cell migration. Suppresses tumor	Human, Mice, Zebrafish, Xenopus laevis	[326,327]

			growth and migration in animal models.		
SDC4 (Syndecan 4)	ribEGC	5DPI	Associated with inflammatory responses; Required for early-stage repair responses during zebrafish heart regeneration; Crucial role played by Cxcl10-Sdc4 in the inflammatory dynamics.	Human, Mice, Zebrafish, Xenopus gastrulae	328,329
RBM10 (RNA binding motif protein 10)	MSN	5DPI	Regulates the alternative splicing of primary transcripts. Association of RBM10 mutations with various cancers. Inhibits cell proliferation and promotes apoptosis (tumor suppressor)	Human, Mice	330
WIPF3 (WAS/WASL Interacting Protein Family Member 3)	MSN	5DPI	May regulate actin dynamics. It is a verprolin family member that forms a complex with N-WASP.	Human	331

Bibliography

1. Sharma, A. V., Ganguly, K., Paul, S., Maulik, N. & Swarnakar, S. Curcumin Heals Indomethacin-Induced Gastric Ulceration by Stimulation of Angiogenesis and Restitution of Collagen Fibers *via* VEGF and MMP-2 Mediated Signaling. *Antioxid Redox Signal* 16, 351–362 (2012).

2. Groft, L. L. *et al.* Differential expression and localization of TIMP-1 and TIMP-4 in human gliomas. *Br J Cancer* 85, 55–63 (2001).

3. Chan, Z. C.-K., Oentaryo, M. J. & Lee, C. W. MMP-mediated modulation of ECM environment during axonal growth and NMJ development. *Neurosci Lett* 724, 134822 (2020).

4. Mendes, O., Kim, H.-T., Lungu, G. & Stoica, G. MMP2 role in breast cancer brain metastasis development and its regulation by TIMP2 and ERK1/2. *Clin Exp Metastasis* 24, 341–351 (2007).

5. Denis, J.-F. *et al.* Activation of Smad2 but not Smad3 is required to mediate TGF-β signaling during axolotl limb regeneration. *Development* 143, 3481–3490 (2016).

6. Saha, S. K. *et al.* KRT19 directly interacts with β-catenin/RAC1 complex to regulate NUMB-dependent NOTCH signaling pathway and breast cancer properties. *Oncogene* 36, 332–349 (2017).

7. Sun, S. *et al.* DLL4 restores damaged liver by enhancing hBMSC differentiation into cholangiocytes. *Stem Cell Res* 47, 101900 (2020).

8. Li, J. *et al.* Apocrine gland damage and the release of specific keratins in early stage indicate the crucial involvement of apocrine glands in hidradenitis suppurativa. *Journal of Investigative Dermatology* https://doi.org/10.1016/j.jid.2024.09.021 (2024) doi:10.1016/j.jid.2024.09.021.

9. Asfaha, S. *et al.* Krt19+/Lgr5− Cells Are Radioresistant Cancer-Initiating Stem Cells in the Colon and Intestine. *Cell Stem Cell* 16, 627–638 (2015).

10. Han, S. *et al.* Nuclear KRT19 is a transcriptional corepressor promoting histone deacetylation and liver tumorigenesis. *Hepatology* https://doi.org/10.1097/HEP.0000000000000875 (2024) doi:10.1097/HEP.0000000000000875.

11. Clayton, S. W. *et al.* Single cell RNA sequencing reveals a shift in cell function and maturation of endogenous and infiltrating cell types in response to acute intervertebral disc injury. Preprint at https://doi.org/10.1101/2024.08.10.607363 (2024).

12. Chen, B. *et al.* KRT18 Modulates Alternative Splicing of Genes Involved in Proliferation and Apoptosis Processes in Both Gastric Cancer Cells and Clinical Samples. *Front Genet* 12, (2021).

13. Liang, X. *et al.* KRT18 regulates trophoblast cell migration and invasion which are essential for embryo implantation. *Reproductive Biology and Endocrinology* 21, 78 (2023).

14. Zhang, J., Hu, S. & Li, Y. KRT18 is correlated with the malignant status and acts as an oncogene in colorectal cancer. *Biosci Rep* 39, (2019).

15. Faissner, A., Roll, L. & Theocharidis, U. Tenascin-C in the matrisome of neural stem and progenitor cells. *Molecular and Cellular Neuroscience* 81, 22–31 (2017).

16. Ohashi, A. *et al.* Tenascin-C-enriched regeneration-specific extracellular matrix guarantees superior muscle regeneration in Ambystoma mexicanum. *Dev Biol* 504, 98–112 (2023).

17. Okada, T. & Suzuki, H. The Role of Tenascin-C in Tissue Injury and Repair After Stroke. *Front Immunol* 11, (2021).

18. Pollen, A. A. *et al.* Molecular Identity of Human Outer Radial Glia during Cortical Development. *Cell* 163, 55–67 (2015).

19. Cai, X. *et al.* Tenascin C+ papillary fibroblasts facilitate neuro-immune interaction in a mouse model of psoriasis. *Nat Commun* 14, 2004 (2023).

20. Ferrucci, V. *et al.* Prune-1 drives polarization of tumor-associated macrophages (TAMs) within the lung metastatic niche in triple-negative breast cancer. *iScience* 24, 101938 (2021).

21. Stanic, K. *et al.* The Reprimo gene family member, reprimo-like (rprml), is required for blood development in embryonic zebrafish. *Sci Rep* 9, 7131 (2019).

22. Figueroa, R. J. *et al.* Reprimo tissue-specific expression pattern is conserved between zebrafish and human. *PLoS One* 12, e0178274 (2017).

23. Han, H., Zhu, W., Lin, T., Liu, C. & Zhai, H. N4BP3 promotes angiogenesis in hepatocellular carcinoma by binding with KAT2B. *Cancer Sci* 113, 3390–3404 (2022).

24. Luo, M. *et al.* N4BP3 promotes breast cancer metastasis via NEDD4-mediated E-cadherin ubiquitination and degradation. *Cancer Lett* 550, 215926 (2022).

25. Wang, Y. *et al.* COL1A2 inhibition suppresses glioblastoma cell proliferation and invasion. *J Neurosurg* 138, 639–648 (2023).

26. Omar, R., Cooper, A., Maranyane, H. M., Zerbini, L. & Prince, S. COL1A2 is a TBX3 target that mediates its impact on fibrosarcoma and chondrosarcoma cell migration. *Cancer Lett* 459, 227–239 (2019).

27. Riquelme-Guzmán, C. *et al.* Postembryonic development and aging of the appendicular skeleton in <scp> *Ambystoma mexicanum* </scp>. *Developmental Dynamics* 251, 1015–1034 (2022).

28. Chen, J. W. & Galloway, J. L. The development of zebrafish tendon and ligament progenitors. *Development* 141, 2035–2045 (2014).

29. Ji, C., Zhao, H., Chen, D., Zhang, H. & Zhao, Y. G. β-propeller proteins WDR45 and WDR45B regulate autophagosome maturation into autolysosomes in neural cells. *Current Biology* 31, 1666-1677.e6 (2021).

30. Ji, C. *et al.* Role of *Wdr45b* in maintaining neural autophagy and cognitive function. *Autophagy* 16, 615–625 (2020).

31. Wu, Z., Gao, Y., Cao, L., Peng, Q. & Yao, X. Purine metabolism-related genes and immunization in thyroid eye disease were validated using bioinformatics and machine learning. *Sci Rep* 13, 18391 (2023).

32. Lin, W. *et al.* Identification of a 6-RBP gene signature for a comprehensive analysis of glioma and ischemic stroke: Cognitive impairment and aging-related hypoxic stress. *Front Aging Neurosci* 14, (2022).

33. Antonacopoulou, A. G. *et al.* POLR2F, ATP6V0A1 and PRNP expression in colorectal cancer: new molecules with prognostic significance? *Anticancer Res* 28, 1221–7 (2008).

34. Boyko, A. I. *et al.* Delayed Impact of 2-Oxoadipate Dehydrogenase Inhibition on the Rat Brain Metabolism Is Linked to Protein Glutarylation. *Front Med (Lausanne)* 9, (2022).

35. Fabrizi, G. M. *et al.* Inherited motor-sensory neuropathy with upper limb predominance associated with the tropomyosin-receptor kinase fused gene. *Neuromuscular Disorders* 30, 227–231 (2020).

36. Xu, Y. *et al.* Liver Regeneration-Related Genes of Nontumor Liver Tissues Predict the Prognosis of Patients with Hepatocellular Carcinoma. *J Hepatocell Carcinoma* Volume 10, 2197–2209 (2023).

37. Kim, H. *et al.* Identification and evaluation of midbrain specific longevity-related genes in exceptionally long-lived but healthy mice. *Front Aging Neurosci* 14, (2023).

38. Cao, Q., Zhang, J. & Zhang, T. AIMP2-DX2 Promotes the Proliferation, Migration, and Invasion of Nasopharyngeal Carcinoma Cells. *Biomed Res Int* 2018, 1–11 (2018).

39. Kim, M. *et al.* Fluorescence-Based Analysis of Noncanonical Functions of Aminoacyl-tRNA Synthetase-Interacting Multifunctional Proteins (AIMPs) in Peripheral Nerves. *Materials* 12, 1064 (2019).

40. Kim, H. *et al.* AIMP2 accumulation in brain leads to cognitive deficits and blood secretion in Parkinson's disease. *J Transl Med* 22, 919 (2024).

41. Zhang, Y. *et al.* ZNF365 promotes stability of fragile sites and telomeres. *Cancer Discov* 3, 798–811 (2013).

42. Urista, J. *et al.* Lack of ZNF365 Drives Senescence and Exacerbates Experimental Lung Fibrosis. *Cells* 11, 2848 (2022).

43. Du, X. *et al.* Reduced Proliferative Capacity and Defense against Staphylococcus aureus in Human Nasal Mucosal Epithelium Lacking ZNF365. *Int Arch Allergy Immunol* 185, 466–479 (2024).

44. Du, X., Zhou, Y., Song, L., Wang, X. & Zhang, S. Zinc finger protein 365 is a new maternal LPS-binding protein that defends zebrafish embryos against gram-negative bacterial infections. *The FASEB Journal* 32, 979–994 (2018).

45. Ozmen Yaylaci, A. & Canbek, M. The role of ubiquitin signaling pathway on liver regeneration in rats. *Mol Cell Biochem* 478, 131–147 (2023).

46. Yan, D. *et al.* The APC/C E3 ligase subunit ANAPC11 mediates FOXO3 protein degradation to promote cell proliferation and lymph node metastasis in urothelial bladder cancer. *Cell Death Dis* 14, 516 (2023).

47. Wang, L. *et al.* Dynamic enhancer interactome promotes senescence and aging. Preprint at https://doi.org/10.1101/2023.05.22.541769 (2023).

48. Wang, R., Zheng, J., Zhang, D.-S., Yang, Y.-H. & Zhao, Z.-F. Wnt1-induced MAFK expression promotes osteosarcoma cell proliferation. *Genetics and Molecular Research* 14, 7315–7325 (2015).

49. Katsuoka, F. & Yamamoto, M. Small Maf proteins (MafF, MafG, MafK): History, structure and function. *Gene* 586, 197–205 (2016).

50. Yang, Z. *et al.* Dysregulated COL3A1 and RPL8, RPS16, and RPS23 in Disc Degeneration Revealed by Bioinformatics Methods. *Spine (Phila Pa 1976)* 40, E745–E751 (2015).

51. van der Spek, A. *et al.* Exome Sequencing Analysis Identifies Rare Variants in ATM and RPL8 That Are Associated With Shorter Telomere Length. *Front Genet* 11, (2020).

52. Fan, S. *et al.* Integrative Multi-Omics Analysis of Identified Ferroptosis-Marker RPL8 as a Candidate Oncogene Correlates with Poor Prognosis and Immune Infiltration in Liver Cancer. *Comb Chem High Throughput Screen* 26, 1298–1310 (2023).

53. Mulder, J. *et al.* Secretagogin is a Ca 2+ -binding protein specifying subpopulations of telencephalic neurons. *Proceedings of the National Academy of Sciences* 106, 22492–22497 (2009).

54. Hao, T. *et al.* Predicting human age by detecting DNA methylation status in hair. *Electrophoresis* 42, 1255–1261 (2021).

55. Vidaki, A. *et al.* DNA methylation-based forensic age prediction using artificial neural networks and next generation sequencing. *Forensic Sci Int Genet* 28, 225–236 (2017).

56. Sharma, A. K., Khandelwal, R. & Sharma, Y. Veiled Potential of Secretagogin in Diabetes: Correlation or Coincidence? *Trends in Endocrinology & Metabolism* 30, 234–243 (2019).

57. Ouyang, S. *et al.* The downregulation of SCGN induced by lipotoxicity promotes NLRP3-mediated β-cell pyroptosis. *Cell Death Discov* 10, 340 (2024).

58. Liu, Z. *et al.* SCGN deficiency is a risk factor for autism spectrum disorder. *Signal Transduct Target Ther* 8, 3 (2023).

59. Liu, Z. *et al.* SCGN deficiency is a risk factor for autism spectrum disorder. *Signal Transduct Target Ther* 8, 3 (2023).

60. Kluth, O. *et al.* Identification of Four Mouse Diabetes Candidate Genes Altering β-Cell Proliferation. *PLoS Genet* 11, e1005506 (2015).

61. Bulfone, A. *et al.* Pcp4l1, a novel gene encoding a Pcp4-like polypeptide, is expressed in specific domains of the developing brain. *Gene Expression Patterns* 4, 297–301 (2004).

62. Micheli, L., Ceccarelli, M., Farioli-Vecchioli, S. & Tirone, F. Control of the Normal and Pathological Development of Neural Stem and Progenitor Cells by the PC3/Tis21/Btg2 and Btg1 Genes. *J Cell Physiol* 230, 2881–2890 (2015).

63. Kim, S. H., Jung, I. R. & Hwang, S. S. Emerging role of anti-proliferative protein BTG1 and BTG2. *BMB Rep* 55, 380–388 (2022).

64. Wenger, Y., Buzgariu, W., Reiter, S. & Galliot, B. Injury-induced immune responses in Hydra. *Semin Immunol* 26, 277–294 (2014).

65. Kulikov, E. I., Malakheeva, L. I. & Komarchev, A. S. The role of BTG1 and BTG2 genes and their effects on insulin in poultry. *Front Physiol* 15, (2024).

66. Wang, H. *et al.* Knockdown of transcription factor forkhead box O3 (FOXO3) suppresses erythroid differentiation in human cells and zebrafish. *Biochem Biophys Res Commun* 460, 923–930 (2015).

67. Saka, Y., Tada, M. & Smith, J. C. A screen for targets of the Xenopus T-box gene Xbra. *Mech Dev* 93, 27–39 (2000).

68. Benjamins, J. W. *et al.* Genomic insights in ascending aortic size and distensibility. *EBioMedicine* 75, 103783 (2022).

69. Khalaji, A. *et al.* A bioinformatics-based study on the Cisplatin-resistant lung cancer cells; what are the orchestrators of this phenom? *Gene* 834, 146668 (2022).

70. Su, P. *et al.* Identification of the Key Genes and Pathways in Esophageal Carcinoma. *Gastroenterol Res Pract* 2016, 1–11 (2016).

71. Lipp, S. N., Jacobson, K. R., Hains, D. S., Schwarderer, A. L. & Calve, S. 3D Mapping Reveals a Complex and Transient Interstitial Matrix During Murine Kidney Development. *Journal of the American Society of Nephrology* 32, 1649–1665 (2021).

72. Paschen, S. A. The role of the TIM8-13 complex in the import of Tim23 into mitochondria. *EMBO J* 19, 6392–6400 (2000).

73. Zhou, S., Han, Y., Yang, R., Pi, X. & Li, J. TIMM13 as a prognostic biomarker and associated with immune infiltration in skin cutaneous melanoma (SKCM). *Front Surg* 9, (2022).

74. Han, Q., Yan, P., Song, R., Liu, F. & Tian, Q. HOXC13-driven TIMM13 overexpression promotes osteosarcoma cell growth. *Cell Death Dis* 14, 398 (2023).

75. Qiu, S., Sun, M., Xu, Y. & Hu, Y. Integrating multi-omics data to reveal the effect of genetic variant rs6430538 on Alzheimer's disease risk. *Front Neurosci* 18, (2024).

76. Li, M.-Y., Shi, Y.-C., Xu, W.-X., Zhao, L. & Zhang, A.-Z. Exploring Cr(VI)-induced blood-brain barrier injury and neurotoxicity in zebrafish and snakehead fish, and inhibiting toxic effects of astaxanthin. *Environmental Pollution* 355, 124280 (2024).

77. Yan, H. *et al.* Functional Study of TMEM163 Gene Variants Associated with Hypomyelination Leukodystrophy. *Cells* 11, 1285 (2022).

78. Domarkienė, I. *et al.* RTN4 AND FBXL17 GENES ARE ASSOCIATED WITH CORONARY HEART DISEASE IN GENOME-WIDE ASSOCIATION ANALYSIS OF LITHUANIAN FAMILIES. *Balkan Journal of Medical Genetics* 16, 17–22 (2013).

79. Guan, Y.-P. *et al.* Breast Cancer Association Studies in a Han Chinese Population using 10 European-ancestry-associated Breast Cancer Susceptibility SNPs. *Asian Pacific Journal of Cancer Prevention* 15, 85–91 (2014).

80. Xiao, G. G. *et al.* Identification of F-box/LLR-repeated protein 17 as potential useful biomarker for breast cancer therapy. *Cancer Genomics Proteomics* 5, 151–60 (2008).

81. Martins-Silva, T. *et al.* Host genetics influences the relationship between the gut microbiome and psychiatric disorders. *Prog Neuropsychopharmacol Biol Psychiatry* 106, 110153 (2021).

82. Pascual-Alonso, A. *et al.* Multi-omics in *MECP2* duplication syndrome patients and carriers. *European Journal of Neuroscience* 60, 4004–4018 (2024).

83. Huang, R. *et al.* Cord blood-derived biologics lead to robust axonal regeneration in benzalkonium chloride-injured mouse corneas by modulating the Il-17 pathway and neuropeptide Y. *Molecular Medicine* 30, 2 (2024).

84. Mondal, C. *et al.* A proliferative to invasive switch is mediated by srGAP1 downregulation through the activation of TGF-β2 signaling. *Cell Rep* 40, 111358 (2022).

85. Gu, X. *et al.* MicroRNA124 Regulated Neurite Elongation by Targeting OSBP. *Mol Neurobiol* 53, 6388–6396 (2016).

86. Zhou, Y., Wohlfahrt, G., Paavola, J. & Olkkonen, V. M. A vertebrate model for the study of lipid binding/transfer protein function: Conservation of OSBP-related proteins between zebrafish and human. *Biochem Biophys Res Commun* 446, 675–680 (2014).

87. Guerri, L. *et al.* Low Dopamine D2 Receptor Expression Drives Gene Networks Related to GABA, cAMP, Growth and Neuroinflammation in Striatal Indirect Pathway Neurons. *Biological Psychiatry Global Open Science* 3, 1104–1115 (2023).

88. Carpenter, M. D. *et al.* Nr4a1 suppresses cocaine-induced behavior via epigenetic regulation of homeostatic target genes. *Nat Commun* 11, 504 (2020).

89. Long, K. L. P. *et al.* Transcriptomic profiles of stress susceptibility and resilience in the amygdala and hippocampus. Preprint at https://doi.org/10.1101/2023.02.08.527777 (2023).

90. Singh, A. *et al.* Demystifying functional role of cocaine- and amphetamine-related transcript (CART) peptide in control of energy homeostasis: A twenty-five year expedition. *Peptides (N.Y.)* 140, 170534 (2021).

91. Opazo, R. *et al.* Fasting Upregulates npy, agrp, and ghsr Without Increasing Ghrelin Levels in Zebrafish (Danio rerio) Larvae. *Front Physiol* 9, (2019).

92. Liu, Y. *et al.* Hypoxia causes mitochondrial dysfunction and brain memory disorder in a manner mediated by the reduction of Cirbp. *Science of The Total Environment* 806, 151228 (2022).

93. Chongsathidkiet, P. & Fecci, P. E. Cold-inducible RNA-binding protein (CIRBP) as a biomarker to predict recurrence of brain metastases. *Neuro Oncol* 23, 1419–1420 (2021).

94. Nakamura, S. *et al.* Suppression of autophagic activity by Rubicon is a signature of aging. *Nat Commun* 10, 847 (2019).

95. Pathak, S., Stewart, W. C. L., Burd, C. E., Hester, M. E. & Greenberg, D. A. Brd2 haploinsufficiency extends lifespan and healthspan in C57B6/J mice. *PLoS One* 15, e0234910 (2020).

96. Branigan, G. L. *et al.* Zebrafish Paralogs brd2a and brd2b Are Needed for Proper Circulatory, Excretory and Central Nervous System Formation and Act as Genetic Antagonists during Development. *J Dev Biol* 9, 46 (2021).

97. Li, X. *et al.* HIF-1-induced mitochondrial ribosome protein L52: a mechanism for breast cancer cellular adaptation and metastatic initiation in response to hypoxia. *Theranostics* 11, 7337–7359 (2021).

98. Lavdovskaia, E. *et al.* Dual function of GTPBP6 in biogenesis and recycling of human mitochondrial ribosomes. *Nucleic Acids Res* 48, 12929–12942 (2020).

99. Diehl, D., Friedmann, A. & Bachmann, H. S. Prenyltransferase gene expression reveals an essential role of prenylation for the inflammatory response in human gingival fibroblasts. *J Periodontol* 94, 1450–1460 (2023).

100. Liu, B., Chen, X., Wang, Z. Q. & Tong, W. M. DNA damage and oxidative injury are associated with hypomyelination in the corpus callosum of newborn Nbn CNS-del mice. *J Neurosci Res* 92, 254–266 (2014).

101. Alfieri, C. *et al.* Molecular basis of APC/C regulation by the spindle assembly checkpoint. *Nature* 536, 431–436 (2016).

102. Khoo, C., Hallquist, N. A., Samuelson, D. A. & Cousins, R. J. Differential expression of cysteine-rich intestinal protein in liver and intestine in CCl4-induced inflammation. *American Journal of Physiology-Gastrointestinal and Liver Physiology* 270, G613–G618 (1996).

103. Hempel, A. & Kühl, S. J. Comparative expression analysis of cysteine-rich intestinal protein family members crip1, 2 and 3 during Xenopus laevis embryogenesis. *Int J Dev Biol* 58, 841–849 (2014).

104. Straubinger, J. *et al.* Amplified pathogenic actions of angiotensin II in cysteine-rich LIM-only protein 4–negative mouse hearts. *The FASEB Journal* 31, 1620–1638 (2017).

105. Zhang, L., Zhu, J.-H., Zhang, X. & Cheng, W.-H. The Thioredoxin-Like Family of Selenoproteins: Implications in Aging and Age-Related Degeneration. *Biol Trace Elem Res* 188, 189–195 (2019).

106. Srivastava, A. C. *et al.* Elimination of human folypolyglutamate synthetase alters programming and plasticity of somatic cells. *The FASEB Journal* 33, 13747–13761 (2019).

107. Nilén, G., Larsson, M., Hyötyläinen, T. & Keiter, S. H. A complex mixture of polycyclic aromatic compounds causes embryotoxic, behavioral, and molecular effects in zebrafish larvae (Danio rerio), and in vitro bioassays. *Science of The Total Environment* 906, 167307 (2024).

108. Song, T., He, N., Hao, Z. & Yang, Y. Upregulation of ENKD1 disrupts cellular homeostasis to promote lymphoma development. *J Cell Physiol* 238, 1308–1323 (2023).

109. Yang, S. *et al.* CYLD Maintains Retinal Homeostasis by Deubiquitinating ENKD1 and Promoting the Phagocytosis of Photoreceptor Outer Segments. *Advanced Science* 11, (2024).

110. Song, T. *et al.* Enkurin domain containing 1 (ENKD1) regulates the proliferation, migration and invasion of non-small cell lung cancer cells. *Asia Pac J Clin Oncol* 18, (2022).

111. Mulligan, M. K. *et al.* Expression, covariation, and genetic regulation of miRNA Biogenesis genes in brain supports their role in addiction, psychiatric disorders, and disease. *Front Genet* 4, (2013).

112. Gao, P. *et al.* Identification of the transcriptome signatures and immune-inflammatory responses in postmenopausal osteoporosis. *Heliyon* 10, e23675 (2024).

113. Hirooka, A. *et al.* The gastrin-releasing peptide/bombesin system revisited by a reverse-evolutionary study considering Xenopus. *Sci Rep* 11, 13315 (2021).

114. Xia, G.-Q., Xu, M., Sun, C., Zhang, Z.-L. & Li, X.-Q. Elevated microRNA-214-3p level ameliorates neuroinflammation after spinal cord ischemia–reperfusion injury by inhibiting Nmb/Cav3.2 pathway. *Int Immunopharmacol* 133, 112031 (2024).

115. Gao, X. *et al.* Cervical cancer-produced neuromedin-B reprograms Schwann cells to initiate perineural invasion. *Cell Death Dis* 15, 636 (2024).

116. Wang, J. *et al.* lncRNA ZNRD1-AS1 promotes malignant lung cell proliferation, migration, and angiogenesis via the miR-942/TNS1 axis and is positively regulated by the m6A reader YTHDC2. *Mol Cancer* 21, 229 (2022).

117. Kilaru, V. *et al.* Genome-wide gene-based analysis suggests an association between Neuroligin 1 (NLGN1) and post-traumatic stress disorder. *Transl Psychiatry* 6, e820–e820 (2016).

118. Wang, Y. *et al.* UBE3B promotes breast cancer progression by antagonizing HIF-2α degradation. *Oncogene* 42, 3394–3406 (2023).

119. Qian, X., Zheng, S. & Yu, Y. CircUBE3B High Expression Participates in Sevoflurane-Induced Human Hippocampal Neuron Injury via Targeting miR-326 and Regulating MYD88 Expression. *Neurotox Res* 41, 16–28 (2023).

120. Marquez, J. *et al.* *DLG5* variants are associated with multiple congenital anomalies including ciliopathy phenotypes. *J Med Genet* 58, 453–464 (2021).

121. Liu, J., Li, J., Ren, Y. & Liu, P. DLG5 in Cell Polarity Maintenance and Cancer Development. *Int J Biol Sci* 10, 543–549 (2014).

122. Chengcheng, L. *et al.* Bioinformatics role of the WGCNA analysis and co-expression network identifies of prognostic marker in lung cancer. *Saudi J Biol Sci* 29, 3519–3527 (2022).

123. Negrey, J. D. *et al.* Transcriptional profiles in olfactory pathway–associated brain regions of African green monkeys: Associations with age and Alzheimer's disease neuropathology. *Alzheimer's & Dementia: Translational Research & Clinical Interventions* 8, (2022).

124. Cheong, A., Lingutla, R. & Mager, J. Expression analysis of mammalian mitochondrial ribosomal protein genes. *Gene Expression Patterns* 38, 119147 (2020).

125. Jun, S. *et al.* Organization of Purkinje cell development by neuronal MEGF11 in cerebellar granule cells. *Cell Rep* 42, 113137 (2023).

126. Chiu, J.-H. *et al.* MEGF11 is related to tumour recurrence in triple negative breast cancer via chemokine upregulation. *Sci Rep* 10, 8060 (2020).

127. Woo, S. H. *et al.* Mapping Novel Immunogenic Epitopes in IgA Nephropathy. *Clinical Journal of the American Society of Nephrology* 10, 372–381 (2015).

128. He, J.-Y., Zhou, X.-Q. & Wang, W.-T. [Mechanism of miRNA-3679 Inhibiting Downstream ZADH2-Target Genes to Promote Hepatocellular Carcinoma Cell Proliferation]. *Sichuan Da Xue Xue Bao Yi Xue Ban* 53, 744–751 (2022).

129. Turpen, J. B., Carlson, D. L. & Huang, C. Cloning and developmental expression of Xenopus Stat1. *Dev Comp Immunol* 25, 219–229 (2001).

130. Yao, H. *et al.* Ablation of endothelial *Atg7* inhibits ischemia-induced angiogenesis by upregulating *Stat1* that suppresses *Hif1a* expression. *Autophagy* 19, 1491–1511 (2023).

131. Wang, X. *et al.* Driving axon regeneration by orchestrating neuronal and non-neuronal innate immune responses via the IFNγ-cGAS-STING axis. *Neuron* 111, 236-255.e7 (2023).

132. Narayanan, R. K. *et al.* Charcot–Marie–tooth disease causing mutation (p.R158H) in pyruvate dehydrogenase kinase 3 (PDK3) affects synaptic transmission, ATP production and causes neurodegeneration in a CMTX6 *C. elegans* model. *Hum Mol Genet* 31, 133–145 (2021).

133. Li, C. *et al.* Loss of sphingosine kinase 2 promotes the expansion of hematopoietic stem cells by improving their metabolic fitness. *Blood* 140, 1686–1701 (2022).

134. Fukuda, R. *et al.* Stimulation of glycolysis promotes cardiomyocyte proliferation after injury in adult zebrafish. *EMBO Rep* 21, (2020).

135. Yin, Y.-H. *et al.* Construction of a chromosome-level genome assembly for genome-wide identification of growth-related quantitative trait loci in *Sinocyclocheilus grahami* (Cypriniformes, Cyprinidae). *Zool Res* 42, 262–266 (2021).

136. Sie, C. P. & Maas, S. Conserved recoding RNA editing of vertebrate C1q-related factor C1QL1. *FEBS Lett* 583, 1171–1174 (2009).

137. Altunay, Z. M. *et al.* *C1ql1* expression in oligodendrocyte progenitor cells promotes oligodendrocyte differentiation. *FEBS J* https://doi.org/10.1111/febs.17256 (2024) doi:10.1111/febs.17256.

138. Joshi, J. C. *et al.* RGS2 is an innate immune checkpoint for TLR4 and Gαq-mediated IFNγ generation and lung injury. Preprint at https://doi.org/10.1101/2023.09.22.559016 (2023).

139. Zhu, C. *et al.* Silencing of RGS2 enhances hippocampal neuron regeneration and rescues depression-like behavioral impairments through activation of cAMP pathway. *Brain Res* 1746, 147018 (2020).

140. Dusonchet, J. *et al.* A Parkinson's disease gene regulatory network identifies the signaling protein RGS2 as a modulator of LRRK2 activity and neuronal toxicity. *Hum Mol Genet* 23, 4887–4905 (2014).

141. Lin, S.-J. *et al.* Regulator of G protein signaling 2 (Rgs2) regulates neural crest development through Pparδ-Sox10 cascade. *Biochimica et Biophysica Acta (BBA) - Molecular Cell Research* 1864, 463–474 (2017).

142. O'Donnell, A. M., Nakamura, H., Tomuschat, C., Marayati, N. F. & Puri, P. Altered expression of KCNG3 and KCNG4 in Hirschsprung's disease. *Pediatr Surg Int* 35, 193–197 (2019).

143. Matsuda, N. & Mishina, M. Identification of chaperonin CCTγ subunit as a determinant of retinotectal development by whole-genome subtraction cloning from zebrafish *no tectal neuron* mutant. *Development* 131, 1913–1925 (2004).

144. Liu, W. *et al.* Suppression of CCT3 inhibits melanoma cell proliferation by downregulating CDK1 expression. *J Cancer* 13, 1958–1971 (2022).

145. Choudhry, M. *et al.* Downregulation of BUD31 Promotes Prostate Cancer Cell Proliferation and Migration via Activation of p-AKT and Vimentin In Vitro. *Int J Mol Sci* 24, 6055 (2023).

146. Qin, J. *et al.* Bud31-mediated alternative splicing is required for spermatogonial stem cell self-renewal and differentiation. *Cell Death Differ* 30, 184–194 (2023).

147. Benedict, B. *et al.* The RECQL helicase prevents replication fork collapse during replication stress. *Life Sci Alliance* 3, e202000668 (2020).

148. Olesen, C. *et al.* Global gene expression analysis in fetal mouse ovaries with and without meiosis and comparison of selected genes with meiosis in the testis. *Cell Tissue Res* 328, 207–221 (2007).

149. Masciadri, B. *et al.* Characterization of the BUD31 gene of Saccharomyces cerevisiae. *Biochem Biophys Res Commun* 320, 1342–1350 (2004).

150. Endoh, M. *et al.* PCGF6-PRC1 suppresses premature differentiation of mouse embryonic stem cells by regulating germ cell-related genes. *Elife* 6, (2017).

151. Zdzieblo, D. *et al.* Pcgf6, a Polycomb Group Protein, Regulates Mesodermal Lineage Differentiation in Murine ESCs and Functions in iPS Reprogramming. *Stem Cells* 32, 3112–3125 (2014).

152. Lan, X. *et al.* PCGF6 controls neuroectoderm specification of human pluripotent stem cells by activating SOX2 expression. *Nat Commun* 13, 4601 (2022).

153. Sørensen, A. T., Rombach, J., Gether, U. & Madsen, K. L. The Scaffold Protein PICK1 as a Target in Chronic Pain. *Cells* 11, 1255 (2022).

154. Xu, J. & Xia, J. Structure and Function of PICK1. *Neurosignals* 15, 190–201 (2006).

155. Metur, S. P. & Klionsky, D. J. SPART links autophagy machinery and lipid droplets in motor neurons. *Autophagy* 19, 2835–2836 (2023).

156. Chou, W.-C. *et al.* Genetic insights into carbohydrate sulfotransferase 8 and its impact on the immunotherapy efficacy of cancer. *Cell Rep* 43, 113641 (2024).

157. Gao, Y. *et al.* Ehbp1 orchestrates orderly sorting of Wnt/Wingless to the basolateral and apical cell membranes. *EMBO Rep* 25, 5053–5079 (2024).

158. Wang, P. *et al.* RAB-10 Promotes EHBP-1 Bridging of Filamentous Actin and Tubular Recycling Endosomes. *PLoS Genet* 12, e1006093 (2016).

159. Feng, P. *et al.* Study on the Reparative Effect of PEGylated Growth Hormone on Ovarian Parameters and Mitochondrial Function of Oocytes From Rats With Premature Ovarian Insufficiency. *Front Cell Dev Biol* 9, (2021).

160. Lamb, C. A. *et al.* <scp>TBC</scp> 1D14 regulates autophagy via the <scp>TRAPP</scp> complex and <scp>ATG</scp> 9 traffic. *EMBO J* 35, 281–301 (2016).

161. Lu, T. *et al.* TBC1D14 inhibits autophagy to suppress lymph node metastasis in head and neck squamous cell carcinoma by downregulating macrophage erythroblast attacher. *Int J Biol Sci* 18, 1795–1812 (2022).

162. Serajee, F. J. Association of INPP1, PIK3CG, and TSC2 gene variants with autistic disorder: implications for phosphatidylinositol signalling in autism. *J Med Genet* 40, 119e–1119 (2003).

163. Benjamin, D. I. *et al.* Inositol Phosphate Recycling Regulates Glycolytic and Lipid Metabolism That Drives Cancer Aggressiveness. *ACS Chem Biol* 9, 1340–1350 (2014).

164. Lin, P. *et al.* Differential requirements for H/ACA ribonucleoprotein components in cell proliferation and response to DNA damage. *Histochem Cell Biol* 144, 543–558 (2015).

165. Pellizzoni, L., Baccon, J., Charroux, B. & Dreyfuss, G. The survival of motor neurons (SMN) protein interacts with the snoRNP proteins fibrillarin and GAR1. *Current Biology* 11, 1079–1088 (2001).

166. Spaulding, E. L., Feidler, A. M., Cook, L. A. & Updike, D. L. RG/RGG repeats in the C. elegans homologs of Nucleolin and GAR1 contribute to sub-nucleolar phase separation. *Nat Commun* 13, 6585 (2022).

167. Li, Z. *et al.* USP25 Inhibits Neuroinflammatory Responses After Cerebral Ischemic Stroke by Deubiquitinating TAB2. *Advanced Science* 10, (2023).

168. Cai, C. *et al.* USP25 regulates KEAP1-NRF2 anti-oxidation axis and its inactivation protects acetaminophen-induced liver injury in male mice. *Nat Commun* 14, 3648 (2023).

169. Tse, W. K. *et al.* Genome-wide loss-of-function analysis of deubiquitylating enzymes for zebrafish development. *BMC Genomics* 10, 637 (2009).

170. Chrystal, P. W. *et al.* The inner junction protein CFAP20 functions in motile and non-motile cilia and is critical for vision. *Nat Commun* 13, 6595 (2022).

171. Hengel, H. *et al.* Bi-allelic loss-of-function variants in BCAS3 cause a syndromic neurodevelopmental disorder. *The American Journal of Human Genetics* 108, 1069–1082 (2021).

172. Nakanoh, S. *et al.* Simultaneous depletion of RB, RBL1 and RBL2 affects endoderm differentiation of human embryonic stem cells. *PLoS One* 17, e0269122 (2022).

173. Chen, J., Xia, P., Liu, Y., Kogan, C. & Cheng, Z. Loss of Rbl2 (Retinoblastoma-Like 2) Exacerbates Myocardial Ischemia/Reperfusion Injury. *J Am Heart Assoc* 11, (2022).

174. Leigh, N. D. *et al.* von Willebrand factor D and EGF domains is an evolutionarily conserved and required feature of blastemas capable of multitissue appendage regeneration. *Evol Dev* 22, 297–311 (2020).

175. Sireci, S. *et al.* HB-EGF promotes progenitor cell proliferation and sensory neuron regeneration in the zebrafish olfactory epithelium. *FEBS J* 291, 2098–2133 (2024).

176. Zhang, Y. *et al.* Novel CH25H+ and OASL+ microglia subclusters play distinct roles in cerebral ischemic stroke. *J Neuroinflammation* 20, 115 (2023).

177. Guan, X. *et al.* A Novel Hybrid of Telmisartan and Borneol Ameliorates Neuroinflammation and White Matter Injury in Ischemic Stroke Through ATF3/CH25H Axis. *Transl Stroke Res* 15, 195–218 (2024).

178. Pereiro, P. *et al.* Interferon-independent antiviral activity of 25-hydroxycholesterol in a teleost fish. *Antiviral Res* 145, 146–159 (2017).

179. Karwacki-Neisius, V. *et al.* WNT signalling control by KDM5C during development affects cognition. *Nature* 627, 594–603 (2024).

180. Kim, Y. *et al.* Physiological effects of KDM5C on neural crest migration and eye formation during vertebrate development. *Epigenetics Chromatin* 11, 72 (2018).

181. Kishita, Y. *et al.* A novel homozygous variant in *MICOS13* / *QIL1* causes hepato-encephalopathy with mitochondrial DNA depletion syndrome. *Mol Genet Genomic Med* 8, (2020).

182. Wang, Y., Dang, H., Qiao, H., Tian, Y. & Guan, Q. PDP1 promotes the progression of breast cancer through STAT3 pathway. *Cell Biochem Funct* 42, (2024).

183. Mazzone, P. *et al.* UBAC1/KPC2 Regulates TLR3 Signaling in Human Keratinocytes through Functional Interaction with the CARD14/CARMA2sh-TANK Complex. *Int J Mol Sci* 21, 9365 (2020).

184. Zhang, Q.-Y. *et al.* Inulin alleviates GenX-induced intestinal injury in mice by modulating the MAPK pathway, cell cycle, and cell adhesion proteins. *Environmental Pollution* 362, 124974 (2024).

185. Bhattamisra, S. K., Koh, H. M., Lim, S. Y., Choudhury, H. & Pandey, M. Molecular and Biochemical Pathways of Catalpol in Alleviating Diabetes Mellitus and Its Complications. *Biomolecules* 11, 323 (2021).

186. Mizuno, F., Barabas, P., Krizaj, D. & Akopian, A. Glutamate-induced internalization of Ca v 1.3 L-type Ca 2+ channels protects retinal neurons against excitotoxicity. *J Physiol* 588, 953–966 (2010).

187. Huang, S. *et al.* Isoliquiritigenin alleviates liver fibrosis through caveolin-1-mediated hepatic stellate cells ferroptosis in zebrafish and mice. *Phytomedicine* 101, 154117 (2022).

188. Feng, Y. *et al.* Widespread transposable element dysregulation in human aging brains with Alzheimer's disease. *Alzheimer's & Dementia* 20, 7495–7517 (2024).

189. Pakzad, B. *et al.* *C1QTNF4* gene p.His198Gln mutation is correlated with early-onset systemic lupus erythematosus in Iranian patients. *Int J Rheum Dis* 23, 1594–1598 (2020).

190. Lin, S.-L. *et al.* Macrophage Wnt7b is critical for kidney repair and regeneration. *Proceedings of the National Academy of Sciences* 107, 4194–4199 (2010).

191. Chen, X. *et al.* α7nAChR activation in AT2 cells promotes alveolar regeneration through WNT7B signaling in acute lung injury. *JCI Insight* 8, (2023).

192. Yin, A., Winata, C. L., Korzh, S., Korzh, V. & Gong, Z. Expression of components of Wnt and Hedgehog pathways in different tissue layers during lung development in Xenopus laevis. *Gene Expression Patterns* 10, 338–344 (2010).

193. Beretta, C. A., Brinkmann, I. & Carl, M. All four zebrafish Wnt7 genes are expressed during early brain development. *Gene Expression Patterns* 11, 277–284 (2011).

194. Garda, A. L., Puelles, L., Rubenstein, J. L. R. & Medina, L. Expression patterns of Wnt8b and Wnt7b in the chicken embryonic brain suggest a correlation with forebrain patterning centers and morphogenesis. *Neuroscience* 113, 689–698 (2002).

195. Kasahara, K. *et al.* EGF receptor kinase suppresses ciliogenesis through activation of USP8 deubiquitinase. *Nat Commun* 9, 758 (2018).

196. Tang, J., Long, G., Xiao, L. & Zhou, L. USP8 positively regulates hepatocellular carcinoma tumorigenesis and confers ferroptosis resistance through β-catenin stabilization. *Cell Death Dis* 14, 360 (2023).

197. Lock, F. E. *et al.* The RASSF8 candidate tumor suppressor inhibits cell growth and regulates the Wnt and NF-κB signaling pathways. *Oncogene* 29, 4307–4316 (2010).

198. Song, Z. *et al.* LncRNA RASSF8-AS1 knockdown displayed antiproliferative and proapoptotic effects through miR-188-3p/ATG7 pathway in ox-LDL-treated vascular smooth muscle cells. *Ann Transl Med* 11, 143–143 (2023).

199. Chino, H., Hatta, T., Natsume, T. & Mizushima, N. Intrinsically Disordered Protein TEX264 Mediates ER-phagy. *Mol Cell* 74, 909-921.e6 (2019).

200. Lascaux, P. *et al.* TEX264 drives selective autophagy of DNA lesions to promote DNA repair and cell survival. *Cell* 187, 5698-5718.e26 (2024).

201. Li, C. *et al.* Megf10 deficiency impairs skeletal muscle stem cell migration and muscle regeneration. *FEBS Open Bio* 11, 114–123 (2021).

202. Boyden, S. E. *et al.* Mutations in the satellite cell gene MEGF10 cause a recessive congenital myopathy with minicores. *Neurogenetics* 13, 115–124 (2012).

203. Tamagnone, L. *et al.* Plexins Are a Large Family of Receptors for Transmembrane, Secreted, and GPI-Anchored Semaphorins in Vertebrates. *Cell* 99, 71–80 (1999).

204. Franzolin, G. *et al.* PlexinB1 Inactivation Reprograms Immune Cells in the Tumor Microenvironment, Inhibiting Breast Cancer Growth and Metastatic Dissemination. *Cancer Immunol Res* 12, 1286–1301 (2024).

205. Park, S., Lee, H., Lee, J., Park, E. & Park, S. Ependymal Cells Require Anks1a for Their Proper Development. *Mol Cells* 42, 245–251 (2019).

206. Zholudeva, A. O. *et al.* The Role of the Adapter Protein Anks1a in the Regulation of Breast Cancer Cell Motility. *Biochemistry (Moscow)* 87, 1651–1661 (2022).

207. Lee, J. *et al.* ANKS1A regulates LDL receptor-related protein 1 (LRP1)-mediated cerebrovascular clearance in brain endothelial cells. *Nat Commun* 14, 8463 (2023).

208. Cajas, Y. N. *et al.* Antioxidant Nobiletin Enhances Oocyte Maturation and Subsequent Embryo Development and Quality. *Int J Mol Sci* 21, 5340 (2020).

209. Hoptak-Solga, A. D. *et al.* Connexin43 (GJA1) is required in the population of dividing cells during fin regeneration. *Dev Biol* 317, 541–548 (2008).

210. Wang, M. *et al.* GDI2 deletion alleviates neurodegeneration and memory loss in the 5xFAD mice model of Alzheimer's disease. *Biochimica et Biophysica Acta (BBA) - Molecular Basis of Disease* 1870, 167093 (2024).

211. You, N. *et al.* COMMD7 functions as molecular target in pancreatic ductal adenocarcinoma. *Mol Carcinog* 56, 607–624 (2017).

212. Kim, Y. *et al.* Physiological effects of KDM5C on neural crest migration and eye formation during vertebrate development. *Epigenetics Chromatin* 11, 72 (2018).

213. Chen, J. *et al.* SAR1B senses leucine levels to regulate mTORC1 signalling. *Nature* 596, 281–284 (2021).

214. Levic, D. S. *et al.* Animal model of Sar1b deficiency presents lipid absorption deficits similar to Anderson disease. *J Mol Med* 93, 165–176 (2015).

215. Doyle, G. A. *et al.* Analysis of LINE-1 Elements in DNA from Postmortem Brains of Individuals with Schizophrenia. *Neuropsychopharmacology* 42, 2602–2611 (2017).

216. Wang, X., Zhang, X.-Y., Liao, N.-Q., He, Z.-H. & Chen, Q.-F. Identification of ribosome biogenesis genes and subgroups in ischaemic stroke. *Front Immunol* 15, (2024).

217. Hsu, C. C., Pai, W. Y., Lai, C. Y., Lu, M. W. & Her, G. M. Genetic characterization and *in vivo* image analysis of novel zebrafish *Danio rerio* pigment mutants. *J Fish Biol* 82, 1671–1683 (2013).

218. Yang, J., He, J., Lu, J. & Jie, L. *Jun, Gal, Cd74, and C1qb* as potential indicator for neuropathic pain. *J Cell Biochem* 119, 4792–4798 (2018).

219. Hu, Y.-L., Pan, X.-M., Xiang, L.-X. & Shao, J.-Z. Characterization of C1q in Teleosts. *Journal of Biological Chemistry* 285, 28777–28786 (2010).

220. Sasako, T. *et al.* Hepatic Sdf2l1 controls feeding-induced ER stress and regulates metabolism. *Nat Commun* 10, 947 (2019).

221. Zhang, L. *et al.* SDF2L1 Inhibits Cell Proliferation, Migration, and Invasion in Nasopharyngeal Carcinoma. *Biomed Res Int* 2020, 1–12 (2020).

222. Wong, M. *et al.* Silencing of <scp>STRN</scp> 4 suppresses the malignant characteristics of cancer cells. *Cancer Sci* 105, 1526–1532 (2014).

223. McKenzie, C. W. *et al.* CFAP54 is required for proper ciliary motility and assembly of the central pair apparatus in mice. *Mol Biol Cell* 26, 3140–3149 (2015).

224. Ferrer, C. M. & Reginato, M. J. Sticking to Sugars at the Metastatic Site: Sialyltransferase ST6GalNAc2 Acts as a Breast Cancer Metastasis Suppressor. *Cancer Discov* 4, 275–277 (2014).

225. Zhao, H. *et al.* ADSCs Promote Tenocyte Proliferation by Reducing the Methylation Level of lncRNA Morf4l1 in Tendon Injury. *Front Chem* 10, (2022).

226. Bugai, A. *et al.* P-TEFb Activation by RBM7 Shapes a Pro-survival Transcriptional Response to Genotoxic Stress. *Mol Cell* 74, 254-267.e10 (2019).

227. Fukushima, K. & Akira, S. Novel insights into the pathogenesis of lung fibrosis: the RBM7–NEAT1–CXCL12–SatM axis at fibrosis onset. *Int Immunol* 33, 659–663 (2021).

228. Burns, D. T. *et al.* Variants in EXOSC9 Disrupt the RNA Exosome and Result in Cerebellar Atrophy with Spinal Motor Neuronopathy. *The American Journal of Human Genetics* 102, 858–873 (2018).

229. Giunta, M. *et al.* Altered RNA metabolism due to a homozygous *RBM7* mutation in a patient with spinal motor neuropathy. *Hum Mol Genet* ddw149 (2016) doi:10.1093/hmg/ddw149.

230. Hung, K.-Y. *et al.* Targeting F-Box Protein Fbxo3 Attenuates Lung Injury Induced by Ischemia-Reperfusion in Rats. *Front Pharmacol* 10, (2019).

231. Gao, Y. *et al.* E3 Ubiquitin Ligase FBXO3 Drives Neuroinflammation to Aggravate Cerebral Ischemia/Reperfusion Injury. *Int J Mol Sci* 23, 13648 (2022).

232. Meng, X. *et al.* MREG suppresses thyroid cancer cell invasion and proliferation by inhibiting Akt-mTOR signaling. *Biochem Biophys Res Commun* 491, 72–78 (2017).

233. Ohbayashi, N., Maruta, Y., Ishida, M. & Fukuda, M. Melanoregulin regulates retrograde melanosome transport through interaction with the RILP·p150Glued complex in melanocytes. *J Cell Sci* https://doi.org/10.1242/jcs.094185 (2012) doi:10.1242/jcs.094185.

234. Takino, J., Miyazaki, S., Nagamine, K. & Hori, T. The Role of RASGRP2 in Vascular Endothelial Cells—A Mini Review. *Int J Mol Sci* 22, 11129 (2021).

235. Nagamine, K., Matsuda, A., Asashima, M. & Hori, T. XRASGRP2 expression during early development of Xenopus embryos. *Biochem Biophys Res Commun* 372, 886–891 (2008).

236. Sato, T., Takino, J., Nagamine, K., Nishio, K. & Hori, T. RASGRP2 Suppresses Apoptosis via Inhibition of ROS Production in Vascular Endothelial Cells. *The Scientific World Journal* 2019, 1–8 (2019).

237. Krasniak, C. S. & Ahmad, S. T. The role of CHMP2BIntron5 in autophagy and frontotemporal dementia. *Brain Res* 1649, 151–157 (2016).

238. Bao, L.-S. & Xia, J.-L. Global analysis of transcriptome sequences highlights accelerated evolution of immune genes in Danio choprae and Danio albolineatus. *Fish Shellfish Immunol* 66, 390–397 (2017).

239. Su Mo, J. & Cheon Chae, S. MicroRNA 452 regulates GTF2E1 expression in colorectal cancer cells. *J Genet* 100, (2021).

240. Yin, Q. *et al.* MiR-155 deficiency protects renal tubular epithelial cells from telomeric and genomic DNA damage in cisplatin-induced acute kidney injury. *Theranostics* 12, 4753–4766 (2022).

241. Goswami, A. K. *et al.* Physio-biochemical and molecular stress regulators and their crosstalk for low-temperature stress responses in fruit crops: A review. *Front Plant Sci* 13, (2022).

242. Xiong, Y. *et al.* Prognostic value of lipid metabolism-related genes in head and neck squamous cell carcinoma. *Immun Inflamm Dis* 9, 196–209 (2021).

243. Qin, J.-Y. *et al.* Unraveling the mechanism of long-term bisphenol S exposure disrupted ovarian lipids metabolism, oocytes maturation, and offspring development of zebrafish. *Chemosphere* 277, 130304 (2021).

244. Hermasch, M. A. *et al.* Evolutionary distinct roles of γ-secretase subunit nicastrin in zebrafish and humans. *J Dermatol Sci* 105, 80–87 (2022).

245. Liu, Z. *et al.* Upregulation of KLHL17 promotes the proliferation and migration of non-small cell lung cancer by activating the Ras/MAPK signaling pathway. *Laboratory Investigation* 102, 1389–1399 (2022).

246. Bassi, M. T. *et al.* SLC7A8, a Gene Mapping within the Lysinuric Protein Intolerance Critical Region, Encodes a New Member of the Glycoprotein-Associated Amino Acid Transporter Family. *Genomics* 62, 297–303 (1999).

247. Asaoka, Y., Nagai, Y., Namae, M., Furutani-Seiki, M. & Nishina, H. SLC7 family transporters control the establishment of left-right asymmetry during organogenesis in medaka by activating mTOR signaling. *Biochem Biophys Res Commun* 474, 146–153 (2016).

248. de Rijk, E., van Strien, F. & Roubos, E. Demonstration of coexisting catecholamine (dopamine), amino acid (GABA), and peptide (NPY) involved in inhibition of melanotrope cell activity in Xenopus laevis: a quantitative ultrastructural, freeze-substitution immunocytochemical study. *The Journal of Neuroscience* 12, 864–871 (1992).

249. Jeong, I. *et al.* mRNA expression and metabolic regulation of npy and agrp1/2 in the zebrafish brain. *Neurosci Lett* 668, 73–79 (2018).

250. Mousley, A., Polese, G., Marks, N. J. & Eisthen, H. L. Terminal Nerve-Derived Neuropeptide Y Modulates Physiological Responses in the Olfactory Epithelium of Hungry Axolotls (*Ambystoma mexicanum*). *The Journal of Neuroscience* 26, 7707–7717 (2006).

251. Subkhangulova, A. *et al.* <scp>SORCS</scp> 1 and <scp>SORCS</scp> 3 control energy balance and orexigenic peptide production. *EMBO Rep* 19, (2018).

252. Lazar, J. *et al.* SORCS1 contributes to the development of renal disease in rats and humans. *Physiol Genomics* 45, 720–728 (2013).

253. Belyaeva, O. V., Lee, S.-A., Adams, M. K., Chang, C. & Kedishvili, N. Y. Short Chain Dehydrogenase/Reductase Rdhe2 Is a Novel Retinol Dehydrogenase Essential for Frog Embryonic Development. *Journal of Biological Chemistry* 287, 9061–9071 (2012).

254. Hong, K., Yang, Q., Yin, H., Zhang, J. & Yu, B. SDR16C5 promotes proliferation and migration and inhibits apoptosis in pancreatic cancer. *Open Life Sci* 18, (2023).

255. Fatemi, S. H., Reutiman, T. J. & Folsom, T. D. The role of lithium in modulation of brain genes: relevance for aetiology and treatment of bipolar disorder. *Biochem Soc Trans* 37, 1090–1095 (2009).

256. Neumann, A. *et al.* Rare variants in IFFO1, DTNB, NLRC3 and SLC22A10 associate with Alzheimer's disease CSF profile of neuronal injury and inflammation. *Mol Psychiatry* 27, 1990–1999 (2022).

257. Fallatah, W. *et al.* A Pex7 Deficient Mouse Series Correlates Biochemical and Neurobehavioral Markers to Genotype Severity—Implications for the Disease Spectrum of Rhizomelic Chondrodysplasia Punctata Type 1. *Front Cell Dev Biol* 10, (2022).

258. Sobolewski, C., Cerella, C., Dicato, M., Ghibelli, L. & Diederich, M. The Role of Cyclooxygenase-2 in Cell Proliferation and Cell Death in Human Malignancies. *Int J Cell Biol* 2010, 1–21 (2010).

259. Xu, L. *et al.* Chenodeoxycholic Acid (CDCA) Promoted Intestinal Epithelial Cell Proliferation by Regulating Cell Cycle Progression and Mitochondrial Biogenesis in IPEC-J2 Cells. *Antioxidants* 11, 2285 (2022).

260. Zhao, H. *et al.* PHLDA1 Blockade Alleviates Cerebral Ischemia/Reperfusion Injury by Affecting Microglial M1/M2 Polarization and NLRP3 Inflammasome Activation. *Neuroscience* 487, 66–77 (2022).

261. Hogan, C. A. *et al.* Expanded <scp>tRNA</scp> methyltransferase family member <scp>TRMT9B</scp> regulates synaptic growth and function. *EMBO Rep* 24, (2023).

262. Zheng, J. *et al.* Microtubule-bundling protein Spef1 enables mammalian ciliary central apparatus formation. *J Mol Cell Biol* 11, 67–77 (2019).

263. Hsieh, M.-C. *et al.* Spinal TNF-α impedes Fbxo45-dependent Munc13-1 ubiquitination to mediate neuropathic allodynia in rats. *Cell Death Dis* 9, 811 (2018).

264. Han, S. *et al.* The E3 Ubiquitin Ligase Protein Associated with Myc (Pam) Regulates Mammalian/Mechanistic Target of Rapamycin Complex 1 (mTORC1) Signaling in Vivo through N- and C-terminal Domains. *Journal of Biological Chemistry* 287, 30063–30072 (2012).

265. Barbieri, A., Carra, S., De Blasio, P., Cotelli, F. & Biunno, I. Sel1l knockdown negatively influences zebrafish embryos endothelium. *J Cell Physiol* 233, 5396–5404 (2018).

266. Ma, S., Ren, N. & Huang, Q. rs10514231 Leads to Breast Cancer Predisposition by Altering ATP6AP1L Gene Expression. *Cancers (Basel)* 13, 3752 (2021).

267. Guo, H. *et al.* Knockdown of HDAC10 inhibits POLE2-mediated DNA damage repair in NSCLC cells by increasing SP1 acetylation levels. *Pulm Pharmacol Ther* 83, 102250 (2023).

268. Gong, X. *et al.* Transcriptome sequencing reveals Gastrodia elata Blume could increase the cell viability of eNPCs under hypoxic condition by improving DNA damage repair ability. *J Ethnopharmacol* 282, 114646 (2022).

269. Lee, Y. J., Han, M.-E., Baek, S.-J., Kim, S.-Y. & Oh, S.-O. MED30 Regulates the Proliferation and Motility of Gastric Cancer Cells. *PLoS One* 10, e0130826 (2015).

270. Sharpe, M. *et al.* Ruvbl2 Suppresses Cardiomyocyte Proliferation During Zebrafish Heart Development and Regeneration. *Front Cell Dev Biol* 10, (2022).

271. Ma, W., Zhang, K., Bao, Z., Jiang, T. & Zhang, Y. SAMD9 Is Relating With M2 Macrophage and Remarkable Malignancy Characters in Low-Grade Glioma. *Front Immunol* 12, (2021).

272. Chefetz, I. *et al.* Normophosphatemic Familial Tumoral Calcinosis Is Caused by Deleterious Mutations in SAMD9, Encoding a TNF-α Responsive Protein. *Journal of Investigative Dermatology* 128, 1423–1429 (2008).

273. Masuda, W. *et al.* TM2D3, a mammalian homologue of Drosophila neurogenic gene product Almondex, regulates surface presentation of Notch receptors. *Sci Rep* 13, 20913 (2023).

274. Wang, N. *et al.* Altered expression of Armet and Mrlp51 in the oocyte, preimplantation embryo, and brain of mice following oocyte in vitro maturation but postnatal brain development and cognitive function are normal. *REPRODUCTION* 142, 401–408 (2011).

275. Huang, T.-N. & Hsueh, Y.-P. Brain-specific transcriptional regulator T-brain-1 controls brain wiring and neuronal activity in autism spectrum disorders. *Front Neurosci* 9, (2015).

276. Liu, Y. *et al.* DNAAF5 promotes hepatocellular carcinoma malignant progression by recruiting USP39 to improve PFKL protein stability. *Front Oncol* 12, (2022).

277. Fink, D. *et al.* Loss of Ing3 Expression Results in Growth Retardation and Embryonic Death. *Cancers (Basel)* 12, 80 (2019).

278. Shah, S., Smith, H., Feng, X., Rancourt, D. E. & Riabowol, K. ING function in apoptosis in diverse model systemsThis paper is one of a selection of papers published in this Special Issue, entitled CSBMCB's 51st Annual Meeting – Epigenetics and Chromatin Dynamics, and has undergone the Journal's usual peer review process. *Biochemistry and Cell Biology* 87, 117–125 (2009).

279. Boyer, N. P., Monkiewicz, C., Menon, S., Moy, S. S. & Gupton, S. L. Mammalian TRIM67 Functions in Brain Development and Behavior. *eNeuro* 5, ENEURO.0186-18.2018 (2018).

280. Anderson, D. *et al.* Zika Virus Changes Methylation of Genes Involved in Immune Response and Neural Development in Brazilian Babies Born With Congenital Microcephaly. *J Infect Dis* 223, 435–440 (2021).

281. Zhang, Y. *et al.* Investigating the shared genetic architecture between anxiety and stroke. *Behavioural Brain Research* 480, 115400 (2025).

282. Chen, Y.-F. *et al.* miR-125b suppresses oral oncogenicity by targeting the anti-oxidative gene PRXL2A. *Redox Biol* 22, 101140 (2019).

283. Li, H. *et al.* Novel degenerative and developmental defects in a zebrafish model of mucolipidosis type IV. *Hum Mol Genet* 26, 2701–2718 (2017).

284. Yamada, S., Kubo, Y., Yamazaki, D., Sekino, Y. & Kanda, Y. Chlorpyrifos inhibits neural induction via Mfn1-mediated mitochondrial dysfunction in human induced pluripotent stem cells. *Sci Rep* 7, 40925 (2017).

285. Aleknonytė-Resch, M. *et al.* Genome-wide case-only analysis of gene-gene interactions with known Parkinson's disease risk variants reveals link between LRRK2 and SYT10. *NPJ Parkinsons Dis* 9, 102 (2023).

286. Woitecki, A. M. H. *et al.* Identification of Synaptotagmin 10 as Effector of NPAS4-Mediated Protection from Excitotoxic Neurodegeneration. *The Journal of Neuroscience* 36, 2561–2570 (2016).

287. Hsu, C. *et al.* Regulation of exosome secretion by Rab35 and its GTPase-activating proteins TBC1D10A–C. *Journal of Cell Biology* 189, 223–232 (2010).

288. Wu, Q., Wang, C., Guo, L., Ge, Q. & Lu, Z. Identification and characterization of novel microRNA candidates from deep sequencing. *Clinica Chimica Acta* 415, 239–244 (2013).

289. Choi, Y. J. *et al.* Therapeutic strategy using novel RET/YES1 dual-target inhibitor in lung cancer. *Biomedicine & Pharmacotherapy* 171, 116124 (2024).

290. Qazi Mohammad Sajid Jamal. Harnessing natural compounds for PIM-1 kinase inhibition: A synergistic approach using virtual screening, molecular dynamics simulations, and free energy calculations. *Cell Mol Biol* 70, 150–159 (2024).

291. Yin, J. *et al.* Inhibition of the Pim1 Oncogene Results in Diminished Visual Function. *PLoS One* 7, e52177 (2012).

292. Yu, H.-C. *et al.* Regulation of Erythroid Differentiation via the HIF1α-NFIL3-PIM1 Signaling Axis Under Hypoxia. *Antioxid Redox Signal* https://doi.org/10.1089/ars.2023.0508 (2024) doi:10.1089/ars.2023.0508.

293. Chen, C.-Y. *et al.* KCNF1 promotes lung cancer by modulating ITGB4 expression. *Cancer Gene Ther* https://doi.org/10.1038/s41417-022-00560-4 (2022) doi:10.1038/s41417-022-00560-4.

294. Yu, X., Wu, Z. & Zhang, N. Machine learning-driven discovery of novel therapeutic targets in diabetic foot ulcers. *Molecular Medicine* 30, 215 (2024).

295. Adler, C. E. *et al.* Abl Family Kinases and Cbl Cooperate with the Nck Adaptor to Modulate Xenopus Development. *Journal of Biological Chemistry* 275, 36472–36478 (2000).

296. Dunbar, E. K. *et al.* Genetics of Constant and Severe Pain in the NAPS2 Cohort of Recurrent Acute and Chronic Pancreatitis Patients. *J Pain* 104754 (2024) doi:10.1016/j.jpain.2024.104754.

297. Li, C. *et al.* Eicosapentaenoic acid-mediated activation of PGAM2 regulates skeletal muscle growth and development via the PI3K/AKT pathway. *Int J Biol Macromol* 268, 131547 (2024).

298. Wisniewska, M. B. *et al.* LEF1/β-Catenin Complex Regulates Transcription of the Cav3.1 Calcium Channel Gene (*Cacna1g*) in Thalamic Neurons of the Adult Brain. *The Journal of Neuroscience* 30, 4957–4969 (2010).

299. Shen, H. *et al.* GRK5 Deficiency in the Hippocampus Leads to Cognitive Impairment via Abnormal Microglial Alterations. *Mol Neurobiol* 60, 1547–1562 (2023).

300. Philipp, M., Berger, I. M., Just, S. & Caron, M. G. Overlapping and Opposing Functions of G Protein-coupled Receptor Kinase 2 (GRK2) and GRK5 during Heart Development. *Journal of Biological Chemistry* 289, 26119–26130 (2014).

301. Jakovcevski, I., Miljkovic, D., Schachner, M. & Andjus, P. R. Tenascins and inflammation in disorders of the nervous system. *Amino Acids* 44, 1115–1127 (2013).

302. Zhou, W. *et al.* The Effects of RBP4 and Vitamin D on the Proliferation and Migration of Vascular Smooth Muscle Cells via the JAK2/STAT3 Signaling Pathway. *Oxid Med Cell Longev* 2022, 1–23 (2022).

303. Chang, J. T., Lehtinen, M. K. & Sive, H. Zebrafish cerebrospinal fluid mediates cell survival through a retinoid signaling pathway. *Dev Neurobiol* 76, 75–92 (2016).

304. Tomić, G. *et al.* Palmitoyl transferase ZDHHC20 promotes pancreatic cancer metastasis. *Cell Rep* 43, 114224 (2024).

305. Huang, X., Wang, M., Zhang, D., Meng, J. & Liu, P. ZDHHC20 Activates AKT Signaling Pathway to Promote Cell Proliferation in Hepatocellular Carcinoma. *J Hepatocell Carcinoma* Volume 11, 1763–1775 (2024).

306. Mommaerts, H., Esguerra, C. V., Hartmann, U., Luyten, F. P. & Tylzanowski, P. Smoc2 modulates embryonic myelopoiesis during zebrafish development. *Developmental Dynamics* 243, 1375–1390 (2014).

307. Irene Díez García-Prieto, I. *et al.* Mutations in the COL18A1 gen associated with knobloch syndrome and structural brain anomalies: a novel case report and literature review of neuroimaging findings. *Neurocase* 28, 11–18 (2022).

308. Bhat, S. *et al.* Mono-allelic KCNB2 variants lead to a neurodevelopmental syndrome caused by altered channel inactivation. *The American Journal of Human Genetics* 111, 761–777 (2024).

309. Fukuda, S., Wu, D. W., Stark, K. & Pelus, L. M. Cloning and Characterization of a Proliferation-Associated Cytokine-Inducible Protein, CIP29. *Biochem Biophys Res Commun* 292, 593–600 (2002).

310. Dai, H., Yan, M. & Li, Y. The zinc-finger protein ZCCHC2 suppresses retinoblastoma tumorigenesis by inhibiting HectH9-mediated K63-linked polyubiquitination and activation of c-Myc. *Biochem Biophys Res Commun* 521, 533–538 (2020).

311. Dobolyi, A. *et al.* Exclusive neuronal expression of SUCLA2 in the human brain. *Brain Struct Funct* 220, 135–151 (2015).

312. Pedrotti, S., Busà, R., Compagnucci, C. & Sette, C. The RNA recognition motif protein RBM11 is a novel tissue-specific splicing regulator. *Nucleic Acids Res* 40, 1021–1032 (2012).

313. Tsai, M.-T. *et al.* Regulation of HGF-induced hepatocyte proliferation by the small GTPase Arf6 through the PIP2-producing enzyme PIP5K1A. *Sci Rep* 7, 9438 (2017).

314. Qiu, J.-Y. *et al.* OXCT1 regulates hippocampal neurogenesis and alleviates cognitive impairment via the Akt/GSK-3β/β-catenin pathway after subarachnoid hemorrhage. *Brain Res* 1827, 148758 (2024).

315. Lord, T. *et al.* A novel high throughput screen to identify candidate molecular networks that regulate spermatogenic stem cell functions. *Biol Reprod* 106, 1175–1190 (2022).

316. Sánchez Carretero, L., Cardeñosa Pérez, À. C., Peces-Barba, G. & Pérez-Rial, S. Differential lung gene expression identified Zscan2 and Bag6 as novel tissue repair players in an experimental COPD model. *PLoS One* 19, e0309166 (2024).

317. Honda, T. & Inui, M. PDZRN3 protects against apoptosis in myoblasts by maintaining cyclin A2 expression. *Sci Rep* 10, 1140 (2020).

318. Gueniot, F. *et al.* Targeting *Pdzrn3* maintains adult blood-brain barrier and central nervous system homeostasis. *Journal of Cerebral Blood Flow & Metabolism* 42, 613–629 (2022).

319. Kuliyev, E. *et al.* Overlapping Role of SCYL1 and SCYL3 in Maintaining Motor Neuron Viability. *The Journal of Neuroscience* 38, 2615–2630 (2018).

320. Sugawara, T., Miura, T., Kawasaki, T., Umezawa, A. & Akutsu, H. The hsa-miR-302 cluster controls ectodermal differentiation of human pluripotent stem cell via repression of DAZAP2. *Regen Ther* 15, 1–9 (2020).

321. Chen, Y. *et al.* Exercise-Induced Reduction of IGF1R Sumoylation Attenuates Neuroinflammation in APP/PS1 Transgenic Mice. *J Adv Res* https://doi.org/10.1016/j.jare.2024.03.025 (2024) doi:10.1016/j.jare.2024.03.025.

322. Yi, J. *et al.* Role of Nesprin-2 and RanBP2 in BICD2-associated brain developmental disorders. *PLoS Genet* 19, e1010642 (2023).

323. Eising, E. *et al.* A set of regulatory genes co-expressed in embryonic human brain is implicated in disrupted speech development. *Mol Psychiatry* 24, 1065–1078 (2019).

324. Yu, Z. *et al.* Sex-Specific Differences in the Transcriptome of the Human Dorsolateral Prefrontal Cortex in Schizophrenia. *Mol Neurobiol* 60, 1083–1098 (2023).

325. Vasileva, N. S. *et al.* Transcriptome Changes in Glioma Cells Cultivated under Conditions of Neurosphere Formation. *Cells* 11, 3106 (2022).

326. Ding, Y. *et al.* Electroacupuncture promotes neurogenesis in the dentate gyrus and improves pattern separation in an early Alzheimer's disease mouse model. *Biol Res* 56, 65 (2023).

327. Abdoli Shadbad, M. *et al.* HSV1 microRNAs in glioblastoma development: an in silico study. *Sci Rep* 14, 27 (2024).

328. Zhu, Y. *et al.* Integrating single-nucleus RNA sequencing and spatial transcriptomics to elucidate a specialized subpopulation of astrocytes, microglia and vascular cells in brains of mouse model of lipopolysaccharide-induced sepsis-associated encephalopathy. *J Neuroinflammation* 21, 169 (2024).

329. Lai, Z.-Y. *et al.* Syndecan-4 is required for early-stage repair responses during zebrafish heart regeneration. *Mol Biol Rep* 51, 604 (2024).

330. De Luca, F., Kha, M., Swärd, K. & Johansson, M. E. Identification of ARMH4 and WIPF3 as human podocyte proteins with potential roles in immunomodulation and cytoskeletal dynamics. *PLoS One* 18, e0280270 (2023).

331. Inoue, A. RBM10: Structure, functions, and associated diseases. *Gene* 783, 145463 (2021).
